# Supplementary material for: Reproductive factors and the risk of incident dementia: A cohort study of UK Biobank participants
Source: PLoS Med. 2022 Apr 5;19(4):e1003955. doi: 10.1371/journal.pmed.1003955 (PMC8982865; doi:10.1371/journal.pmed.1003955)
Supplement: S1 Table — aCollected in women who indicated that they ever had given birth to more than 1 child (N = 184,876). bCollected in women who indicated that they ever had been pregnant (N = 231,352). cCollected in women who indicated that their periods had stopped (had natural menopause) (N = 165,301). dCollected in women who indicated that they had taken the contraceptive pill (N = 220,344). eCollected in women who indicated that they had ever used HRT (N = 104,133). fCollected in women who indicated that they had ever used HRT and not currently using HRT (N = 87,413). HRT, hormone replacement therapy. (DOCX) [file pmed.1003955.s002.docx]

**S1 Table: Number of missingness for each reproductive factor of interest in the UK Biobank.**

| **Reproductive factor** | **Women**  **(N=273 240)** | **Men**  **(N=228 957)** |
| --- | --- | --- |
|  | **N (%)** | **N (%)** |
| Age at menarche | 8 840 (3.2) | - |
| Ever been pregnant | 788 (0.3) | - |
| Number of children | 828 (0.3) | 4 271 (1.9) |
| Age at first live birth ^a^ | 867 (0.5) | - |
| Number of miscarriages ^b^ | 4 827 (2.1) | - |
| Number of stillbirths ^b^ | 4 636 (2.0) | - |
| Number of abortions ^b^ | 5 266 (2.3) | - |
| Reproductive years ^c^ | 32 628 (19.7) | - |
| Age at natural menopause ^c^ | 29 619 (17.9) | - |
| Hysterectomy | 1 000 (0.4) | - |
| Oophorectomy | 4 530 (1.7) | - |
| Ever taken oral contraceptive pills | 1 416 (0.5) | - |
| Age started oral contraceptive pills ^d^ | 8 657 (3.9) | - |
| Ever used HRT | 1 556 (5.7) | - |
| Age started HRT ^e^ | 12 511 (12.0) | - |
| Duration of HRT use ^f^ | 14 283 (16.3) | - |

HRT, Hormone Replacement Therapy.

^a^ Collected in women who indicated that they ever had given birth to more than one child (N = 184 876).

^b^ Collected in women who indicated that they ever had been pregnant (N = 231 352).

^c^ Collected in women who indicated that their periods had stopped (had natural menopause) (N = 165 301).

^d^ Collected in women who indicated that they had taken the contraceptive pill (N = 220 344).

^e^ Collected in women who indicated that they had ever used HRT (N = 104 133).

^f^ Collected in women who indicated that they had ever used HRT and not currently using HRT (N = 87 413).
