# Supplementary material for: Reproductive factors and the risk of incident dementia: A cohort study of UK Biobank participants
Source: PLoS Med. 2022 Apr 5;19(4):e1003955. doi: 10.1371/journal.pmed.1003955 (PMC8982865; doi:10.1371/journal.pmed.1003955)
Supplement: S2 Table — aAnalyses were adjusted for age, Townsend index, ethnicity, smoking status, systolic blood pressure, BMI, diabetes, total cholesterol, antihypertensive drugs, and lipid-lowering drug. BMI, body mass index; CI, confidence interval; HR, hazard ratio; RHR, ratio of hazard ratio. (DOCX) [file pmed.1003955.s003.docx]

**S2 Table: Unadjusted and multiple-adjusted hazard ratios, and women-to-men ratio of hazard ratios (95% confidence intervals) for the risk of dementia associated with number of children for women and men.**

|  | **Women** | | | | | **Men** | | | | | **Women-to-men** | | | |
| --- | --- | --- | --- | --- | --- | --- | --- | --- | --- | --- | --- | --- | --- | --- |
| **Number of children** | **No of events** | **Unadjusted HR**  **(95% CI)** | **P-value** | **Multiple-adjusted HR**  **(95% CI) ^a^** | **P-value** | **No of events** | **Unadjusted HR**  **(95% CI)** | **P-value** | **Multiple-adjusted HR**  **(95% CI) ^a^** | **P-value** | **Unadjusted RHR**  **(95% CI)** | **P-value** | **Adjusted RHR**  **(95% CI) ^a^** | **P-value** |
| 0 | 284 | 0.86  (0.75, 0.98) | 0.338 | 1.18  (1.04, 1.33) | 0.027 | 363 | 0.84  (0.74, 0.94) | 0.005 | 1.10  (0.98, 1.23) | 0.164 | 1.03  (0.88, 1.20) | 0.760 | 1.09  (0.92, 1.28) | 0.403 |
| 1 | 218 | 0.93  (0.79, 1.06) | 0.312 | 1.09  (0.95, 1.25) | 0.276 | 237 | 0.89  (0.77, 1.02) | 0.126 | 0.97  (0.84, 1.11) | 0.696 | 1.04  (0.86, 1.24) | 0.745 | 1.14  (0.94, 1.39) | 0.242 |
| 2 (ref) | 773 | 1.00  (0.93, 1.07) | - | 1.00  (0.93, 1.08) | - | 876 | 1.00  (0.93, 1.07) | - | 1.00  (0.93, 1.07) | - | 1.00  (0.91, 1.10) | - | 1.00  (0.90, 1.11) | - |
| 3 | 392 | 1.25  (1.15, 1.35) | <0.001 | 1.03  (0.93, 1.15) | 0.641 | 421 | 1.19  (1.10, 1.29) | 0.003 | 1.08  (0.97, 1.19) | 0.237 | 1.05  (0.91, 1.20) | 0.577 | 0.96  (0.83, 1.12) | 0.692 |
| 4 or more | 190 | 1.69  (1.55, 1.83) | <0.001 | 1.14  (0.98, 1.33) | 0.132 | 225 | 1.49  (1.36, 1.62) | <0.001 | 1.26  (1.10, 1.45) | 0.003 | 1.13  (0.93, 1.37) | 0.258 | 0.93  (0.76, 1.14) | 0.530 |

CI, Confidence Intervals; HR, Hazard Ratio; RHR, Ratio of Hazard Ratios.

^a^ Analyses were adjusted for age, Townsend index, ethnicity, smoking status, systolic blood pressure, body mass index, diabetes, total cholesterol, antihypertensive drugs, lipids lowering drug.
