# Supplementary material for: Reproductive factors and the risk of incident dementia: A cohort study of UK Biobank participants
Source: PLoS Med. 2022 Apr 5;19(4):e1003955. doi: 10.1371/journal.pmed.1003955 (PMC8982865; doi:10.1371/journal.pmed.1003955)
Supplement: S3 Table — aEarly menarche was defined as age at first menstrual period before the age of 12 years. bEach live birth in women and each child fathered in men. cEarly menopause was defined as the permanent absence of menstrual periods before the age of 47 years. BMI, body mass index; CI, confidence interval; HR, hazard ratio; HRT, hormone replacement therapy; SES, socioeconomic status. (DOCX) [file pmed.1003955.s004.docx]

| **Reproductive factor** | **Age** | | | | **Socioeconomic status** | | | |
| --- | --- | --- | --- | --- | --- | --- | --- | --- |
|  | **<65 years**  **HR (95% CI)** | **≥65 years**  **HR (95% CI)** | **P-value** | **Higher SES**  **HR (95% CI)** | | **Lower SES**  **HR (95% CI)** | **P-value** |  |
| Early menarche ^a^ vs. not | 1.22 (1.03, 1.44) | 1.11 (0.95, 1.30) | 0.442 | 1.13 (0.98, 1.31) | | 1.03 (0.83, 1.27) | 0.440 |  |
| Age at first live birth per year | 0.94 (0.92, 0.95) | 0.98 (0.96, 1.00) | <0.001 | 0.95 (0.94, 0.96) | | 0.94 (0.93, 0.96) | 0.651 |  |
| Each child ^b^ |  |  |  |  | |  |  |  |
| Women | 1.10 (1.04, 1.16) | 1.02 (0.97, 1.07) | 0.058 | 1.08 (1.02, 1.13) | | 1.22 (1.16, 1.28) | <0.001 |  |
| Men | 1.05 (1.03, 1.07) | 1.02 (0.98, 1.07) | 0.210 | 1.11 (1.07, 1.15) | | 1.05 (1.03, 1.07) | 0.007 |  |
| Stillbirth vs. not | 1.46 (0.99, 2.14) | 1.32 (0.99, 1.76) | 0.690 | 1.53 (1.11, 2.09) | | 1.58 (1.08, 2.30) | 0.890 |  |
| Miscarriage vs. not | 0.91 (0.76, 1.09) | 0.97 (0.83, 1.13) | 0.609 | 0.87 (0.75, 1.02) | | 0.95 (0.77, 1.18) | 0.528 |  |
| Abortion vs. not | 0.62 (0.50, 0.79) | 1.00 (0.80, 1.25) | 0.005 | 0.55 (0.44, 0.69) | | 0.62 (0.48, 0.80) | 0.510 |  |
| Early menopause‡ vs. not | 1.44 (1.16, 1.78) | 1.42 (1.17, 1.72) | 0.946 | 1.28 (1.06, 1.54) | | 1.64 (1.27, 2.11) | 0.123 |  |
| Hysterectomy vs. not | 1.84 (1.58, 2.15) | 1.10 (0.97, 1.26) | <0.001 | 1.55 (1.36, 1.76) | | 2.10 (1.75, 2.51) | 0.008 |  |
| Oophorectomy vs. not | 1.78 (1.44, 2.19) | 1.03 (0.85, 1.25) | <0.001 | 1.23 (1.01, 1.49) | | 2.16 (1.71, 2.73) | <0.001 |  |
| Oral contraceptive pill use vs. not | 0.44 (0.38, 0.51) | 0.89 (0.79, 1.00) | <0.001 | 0.41 (0.36, 0.46) | | 0.42 (0.35, 0.50) | 0.831 |  |
| HRT use vs. not | 2.25 (1.96, 2.59) | 0.86 (0.76, 0.97) | <0.001 | 1.87 (1.66, 2.10) | | 2.07 (1.75, 2.45) | 0.335 |  |
|  | | | | | | | | |
| **Reproductive factor** | **Smoking status** | | | | **BMI** | | | |
|  | **Never**  **HR (95% CI)** | **Ever**  **HR (95% CI)** | **P-value** | **≤25 kg/m^2^**  **HR (95% CI)** | | **>25 kg/m^2^**  **HR (95% CI)** | **P-value** |  |
| Early menarche ^a^ vs. not | 1.07 (0.92, 1.25) | 1.17 (0.99, 1.38) | 0.438 | 1.27 (1.04, 1.56) | | 1.04 (0.90, 1.19) | 0.106 |  |
| Age at first live birth per year | 0.94 (0.92, 0.95) | 0.95 (0.94, 0.97) | 0.069 | 0.94 (0.92, 0.95) | | 0.95 (0.94, 0.96) | 0.283 |  |
| Each child ^b^ |  |  |  |  | |  |  |  |
| Women | 1.12 (1.07, 1.17) | 1.18 (1.12, 1.25) | 0.142 | 1.15 (1.07, 1.22) | | 1.14 (1.10, 1.19) | 0.956 |  |
| Men | 1.07 (1.04, 1.10) | 1.05 (1.04, 1.07) | 0.310 | 1.14 (1.09, 1.18) | | 1.06 (1.04, 1.07) | <0.001 |  |
| Stillbirth vs. not | 1.45 (1.03, 2.03) | 1.76 (1.28, 2.42) | 0.411 | 1.70 (1.11, 2.60) | | 1.57 (1.19, 2.06) | 0.752 |  |
| Miscarriage vs. not | 0.78 (0.66, 0.93) | 1.01 (0.85, 1.19) | 0.039 | 0.74 (0.60, 0.92) | | 0.98 (0.85, 1.13) | 0.036 |  |
| Abortion vs. not | 0.66 (0.52, 0.83) | 0.52 (0.42, 0.65) | 0.154 | 0.65 (0.50, 0.84) | | 0.58 (0.47, 0.71) | 0.486 |  |
| Early menopause ^c^ vs. not | 1.33 (1.08, 1.64) | 1.53 (1.25, 1.86) | 0.347 | 1.41 (1.11, 1.79) | | 1.41 (1.17, 1.69) | 0.990 |  |
| Hysterectomy vs. not | 1.99 (1.74, 2.28) | 1.50 (1.29, 1.75) | 0.007 | 1.76 (1.46, 2.11) | | 1.76 (1.56, 1.98) | 0.996 |  |
| Oophorectomy vs. not | 1.77 (1.47, 2.14) | 1.32 (1.06, 1.64) | 0.044 | 1.61 (1.24, 2.09) | | 1.51 (1.27, 1.79) | 0.687 |  |
| Oral contraceptive pill use vs. not | 0.39 (0.35, 0.44) | 0.40 (0.35, 0.47) | 0.762 | 0.37 (0.32, 0.44) | | 0.44 (0.39, 0.49) | 0.111 |  |
| HRT use vs. not | 1.83 (1.62, 2.08) | 1.99 (1.73, 2.28) | 0.406 | 1.99 (1.71, 2.32) | | 1.87 (1.66, 2.09) | 0.506 |  |

**S3 Table: Unadjusted hazard ratios (95% confidence intervals) for the risk of dementia associated with reproductive factors, by age, SES, smoking, BMI.**

BMI, Body Mass Index; CI, Confidence Intervals; HR, Hazard Ratio; HRT, Hormone Replacement Therapy; SES, Socioeconomic Status.

^a^ Early menarche was defined as age at first menstrual period before the age of 12 years.

^b^ Each livebirth in women and each child fathered in men.

^c^ Early menopause was defined as the permanent absence of menstrual periods before the age of 47 years.
