# Supplementary material for: Reproductive factors and the risk of incident dementia: A cohort study of UK Biobank participants
Source: PLoS Med. 2022 Apr 5;19(4):e1003955. doi: 10.1371/journal.pmed.1003955 (PMC8982865; doi:10.1371/journal.pmed.1003955)
Supplement: S4 Table — aAnalyses were adjusted for age, Townsend index, ethnicity, smoking status, systolic blood pressure, BMI, diabetes, total cholesterol, antihypertensive drugs, and lipid-lowering drugs. BMI, body mass index; CI, confidence interval; HR, hazard ratio; SES, socioeconomic status. (DOCX) [file pmed.1003955.s005.docx]

**S4 Table: Unadjusted and multiple-adjusted hazard ratios for the risk of dementia associated with history and timing of hysterectomy and oophorectomy.**

| **Procedure(s)** | **No of events** | **Unadjusted HR**  **(95% CI)** | **P-value** | **Multiple-adjusted HR**  **(95% CI) ^a^** | **P-value** |
| --- | --- | --- | --- | --- | --- |
| No hysterectomy or oophorectomy (ref) | 1301 | 1.00 (0.95, 1.05) | - | 1.00 (0.94, 1.06) | - |
| Oophorectomy only | 9 | 1.55 (0.90, 2.21) | 0.188 | 1.00 (0.48, 2.09) | 0.996 |
| Hysterectomy only | 279 | 1.75 (1.63, 1.87) | <0.001 | 1.13 (1.00, 1.28) | 0.074 |
| Hysterectomy after oophorectomy | 6 | 3.99 (3.19, 4.79) | <0.001 | 2.35 (1.06, 5.23) | 0.037 |
| Hysterectomy with oophorectomy | 160 | 1.49 (1.33, 1.64) | <0.001 | 0.96 (0.82, 1.14) | 0.676 |
| Hysterectomy before oophorectomy | 17 | 1.96 (1.48, 2.44) | 0.006 | 1.26 (0.76, 2.08) | 0.381 |

CI, Confidence Intervals.

^a^ Analyses were adjusted for age, Townsend index, ethnicity, smoking status, systolic blood pressure, body mass index, diabetes, total cholesterol, antihypertensive drugs, lipids lowering drugs.
