# Supplementary material for: Reproductive factors and the risk of incident dementia: A cohort study of UK Biobank participants
Source: PLoS Med. 2022 Apr 5;19(4):e1003955. doi: 10.1371/journal.pmed.1003955 (PMC8982865; doi:10.1371/journal.pmed.1003955)
Supplement: S5 Table — aAnalyses were adjusted for age, Townsend index, ethnicity, smoking status, systolic blood pressure, BMI, diabetes, total cholesterol, antihypertensive drugs, and lipid-lowering drugs. BMI, body mass index; CI, confidence interval; HR, hazard ratio; HRT, hormone replacement therapy. (DOCX) [file pmed.1003955.s006.docx]

**S5 Table: Unadjusted and multiple-adjusted hazard ratios (95% confidence intervals) for the risk of dementia associated with the timing of HRT use in relation to age at (natural or artificial) menopause in postmenopausal women.**

| **Reproductive factor** | **No of events** | **Unadjusted HR**  **(95% CI)** | **P-value** | **Multiple-adjusted HR**  **(95% CI) ^a^** | **P-value** |
| --- | --- | --- | --- | --- | --- |
| No HRT use (ref) | 771 | 1.00 (0.93, 1.07) | - | 1.00 (0.93, 1.08) | - |
| HRT use, unknown initiation | 253 | 2.23 (1.97, 2.52) | <0.001 | 1.49 (1.31, 1.69) | <0.001 |
| HRT use initiated before menopause | 255 | 1.08 (0.96, 1.22) | 0.278 | 0.89 (0.78, 1.01) | 0.122 |
| HRT use initiation at the same time as menopause | 197 | 0.94 (0.82, 1.08) | 0.458 | 0.86 (0.75, 1.00) | 0.085 |
| HRT use initiated after menopause | 262 | 1.15 (1.02, 1.29) | 0.057 | 0.90 (0.79, 1.02) | 0.149 |

CI, Confidence Intervals; HRT, Hormone Replacement Therapy.

^a^ Analyses were adjusted for age, Townsend index, ethnicity, smoking status, systolic blood pressure, body mass index, diabetes, total cholesterol, antihypertensive drugs, lipids lowering drug.
