# Supplementary material for: Reproductive factors and the risk of incident dementia: A cohort study of UK Biobank participants
Source: PLoS Med. 2022 Apr 5;19(4):e1003955. doi: 10.1371/journal.pmed.1003955 (PMC8982865; doi:10.1371/journal.pmed.1003955)
Supplement: S6 Table — aAnalyses were adjusted for age, Townsend index, ethnicity, smoking status, systolic blood pressure, BMI, diabetes, total cholesterol, antihypertensive drugs, and lipid-lowering drugs. BMI, body mass index; CI, confidence interval; HR, hazard ratio. (DOCX) [file pmed.1003955.s007.docx]

**S6 Table: Unadjusted and multiple-adjusted hazard ratios (95% confidence intervals) for the risk of dementia associated with age at menarche, reproductive years and age at menopause, after excluding those had hysterectomy or oophorectomy.**

| **Reproductive factor** | **No. of events** | **Unadjusted**  **HR (95% CI)** | **P-value** | **Multiple-adjusted HR (95% CI) ^a^** | **P-value** |
| --- | --- | --- | --- | --- | --- |
| Age at menarche |  |  |  |  |  |
| <12 | 258 | 1.27 (1.13, 1.44) | <0.001 | 1.22 (1.08, 1.39) | 0.002 |
| 12 | 228 | 1.12 (0.99, 1.28) | 0.083 | 1.05 (0.91, 1.21) | 0.512 |
| 13 (ref) | 267 | 1.00 (0.89, 1.13) | - | 1.00 (0.88, 1.13) | - |
| 14 | 234 | 1.09 (0.96, 1.24) | 0.188 | 0.94 (0.82, 1.08) | 0.385 |
| >14 | 250 | 1.39 (1.23, 1.57) | <0.001 | 1.26 (1.10, 1.43) | <0.001 |
| Reproductive years |  |  |  |  |  |
| <33 (ref) | 186 | 1.00 (0.87, 1.15) | - | 1.00 (0.86, 1.17) | - |
| 33-35 | 178 | 0.88 (0.76, 1.02) | 0.088 | 0.98 (0.84, 1.15) | 0.813 |
| 36-37 | 166 | 0.71 (0.61, 0.83) | <0.001 | 0.78 (0.67, 0.92) | 0.002 |
| 38-39 | 160 | 0.60 (0.52, 0.71) | <0.001 | 0.67 (0.57, 0.79) | <0.001 |
| 40-42 | 192 | 0.68 (0.59, 0.78) | <0.001 | 0.68 (0.58, 0.79) | <0.001 |
| >42 | 137 | 0.90 (0.76, 1.07) | 0.229 | 0.80 (0.67, 0.95) | 0.012 |
| Age at menopause |  |  |  |  |  |
| <47 | 242 | 1.28 (1.13, 1.46) | <0.001 | 1.32 (1.15, 1.51) | <0.001 |
| 47-49 | 161 | 0.91 (0.78, 1.06) | 0.230 | 1.07 (0.91, 1.26) | 0.423 |
| 50 (ref) | 180 | 1.00 (0.86, 1.16) | - | 1.00 (0.86, 1.17) | - |
| 51-52 | 173 | 0.76 (0.66, 0.88) | <0.001 | 0.80 (0.68, 0.94) | 0.007 |
| 53-54 | 107 | 0.70 (0.58, 0.84) | <0.001 | 0.76 (0.62, 0.93) | 0.008 |
| >54 | 193 | 1.10 (0.96, 1.27) | 0.183 | 0.93 (0.80, 1.08) | 0.349 |

HR, Hazard Ratio; CI, Confidence Intervals.

^a^ Analyses were adjusted for age, Townsend index, ethnicity, smoking status, systolic blood pressure, body mass index, diabetes, total cholesterol, antihypertensive drugs, lipids lowering drugs.
