# Supplementary material for: Reproductive factors and the risk of incident dementia: A cohort study of UK Biobank participants
Source: PLoS Med. 2022 Apr 5;19(4):e1003955. doi: 10.1371/journal.pmed.1003955 (PMC8982865; doi:10.1371/journal.pmed.1003955)
Supplement: S7 Table — Analyses were adjusted for age, Townsend index, ethnicity, smoking status, systolic blood pressure, BMI, diabetes, total cholesterol, antihypertensive drugs, and lipid-lowering drugs. aThese CIs were calculated without the floating absolute risk, to provide comparable results to the competing risk models and the multinomial regression models. BMI, body mass index; CI, confidence interval; HR, hazard ratio; HRT, hormone replacement therapy; OR, odds ratio. (DOCX) [file pmed.1003955.s008.docx]

**S7 Table: Multiple-adjusted Risk Ratios (95% confidence intervals) for the risk of dementia associated with reproductive factors, comparing Cox Proportional Hazards Regression (Hazard Ratios), Competing Risk Model (Hazard Ratios), and Multinomial Regression (Odds Ratios).**

| **Reproductive factor** | **Cox Regression Model ^a^**  **HR**  **(95% CI)** | **P-value** | **Fine and Gray Competing Risk Model**  **Sub distribution HR**  **(95% CI)** | **P-value** | **Multinomial Regression Model**  **OR**  **(95% CI)** | **P-value** |
| --- | --- | --- | --- | --- | --- | --- |
| Age at menarche |  |  |  |  |  |  |
| <12 | 1.20 (1.03, 1.40) | 0.020 | 1.20 (1.03, 1.40) | 0.020 | 1.20 (1.03, 1.40) | 0.020 |
| 12 | 1.07 (0.92, 1.26) | 0.406 | 1.07 (0.91, 1.25) | 0.411 | 1.07 (0.92, 1.26) | 0.406 |
| 13 (ref) | - | - | - |  | - | - |
| 14 | 0.97 (0.83, 1.14) | 0.720 | 0.98 (0.83, 1.14) | 0.815 | 0.98 (0.83, 1.15) | 0.820 |
| >14 | 1.19 (1.02, 1.40) | 0.031 | 1.20 (1.03, 1.40) | 0.020 | 1.21 (1.04, 1.42) | 0.016 |
| Number of live births |  |  |  |  |  |  |
| 0 | 1.18 (1.02, 1.36) | 0.024 | 1.16 (1.01, 1.34) | 0.039 | 1.19 (1.03, 1.38) | 0.020 |
| 1 | 1.09 (0.93, 1.28) | 0.294 | 1.09 (0.93, 1.28) | 0.294 | 1.10 (0.94, 1.29) | 0.240 |
| 2 (ref) | - | - | - | - | - | - |
| 3 | 1.03 (0.91, 1.18) | 0.669 | 1.04 (0.91, 1.18) | 0.566 | 1.04 (0.91, 1.19) | 0.578 |
| 4 or more | 1.14 (0.96, 1.36) | 0.141 | 1.14 (0.96, 1.36) | 0.141 | 1.17 (0.99, 1.40) | 0.075 |
| Age at first live birth |  |  |  |  |  |  |
| <21 | 1.43 (1.18, 1.73) | <0.001 | 1.45 (1.20, 1.74) | <0.001 | 1.45 (1.20, 1.75) | <0.001 |
| 21-22 | 1.23 (1.02, 1.49) | 0.032 | 1.24 (1.02, 1.50) | 0.029 | 1.25 (1.03, 1.51) | 0.022 |
| 23-24 | 1.26 (1.05, 1.52) | 0.014 | 1.26 (1.05, 1.52) | 0.014 | 1.27 (1.05, 1.53) | 0.013 |
| 25-26 (ref) | - | - | - | - | - | - |
| 27-29 | 1.16 (0.96, 1.41) | 0.130 | 1.16 (0.96, 1.40) | 0.123 | 1.15 (0.95, 1.40) | 0.158 |
| >29 | 1.11 (0.91, 1.36) | 0.313 | 1.11 (0.91, 1.36) | 0.313 | 1.10 (0.90, 1.35) | 0.363 |
| Number of miscarriages |  |  |  |  |  |  |
| 0 (ref) | - | - | - | - | - | - |
| 1 | 0.90 (0.77, 1.04) | 0.170 | 0.90 (0.77, 1.04) | 0.170 | 0.89 (0.77, 1.04) | 0.129 |
| 2 or more | 1.02 (0.83, 1.26) | 0.863 | 1.02 (0.83, 1.26) | 0.863 | 1.03 (0.84, 1.28) | 0.796 |
| Number of stillbirths |  |  |  |  |  |  |
| 0 (ref) | - | - | - | - | - | - |
| 1 | 1.15 (0.88, 1.50) | 0.309 | 1.13 (0.86, 1.48) | 0.384 | 1.15 (0.88, 1.51) | 0.315 |
| 2 or more | 1.27 (0.66, 2.45) | 0.485 | 1.23 (0.64, 2.37) | 0.546 | 1.29 (0.66, 2.51) | 0.464 |
| Number of abortions |  |  |  |  |  |  |
| 0 (ref) | - |  | - |  | - |  |
| 1 | 0.97 (0.81, 1.16) | 0.753 | 0.98 (0.83, 1.18) | 0.833 | 0.95 (0.79, 1.14) | 0.596 |
| 2 or more | 0.34 (0.18, 0.64) | <0.001 | 0.36 (0.91, 0.67) | <0.001 | 0.33 (0.18, 0.62) | <0.001 |
| Reproductive years |  |  |  |  |  |  |
| <33 (ref) | - | - | - | - | - | - |
| 33-35 | 0.98 (0.79, 1.22) | 0.865 | 0.98 (0.79, 1.23) | 0.868 | 0.98 (0.78, 1.23) | 0.872 |
| 36-37 | 0.78 (0.62, 0.98) | 0.033 | 0.78 (0.62, 0.97) | 0.029 | 0.77 (0.61, 0.97) | 0.027 |
| 38-39 | 0.67 (0.54, 0.84) | <0.001 | 0.67 (0.53, 0.83) | <0.001 | 0.66 (0.53, 0.83) | <0.001 |
| 40-42 | 0.68 (0.55, 0.85) | <0.001 | 0.67 (0.54, 0.84) | <0.001 | 0.67 (0.54, 0.83) | <0.001 |
| >42 | 0.80 (0.63, 1.01) | 0.063 | 0.78 (0.62, 0.99) | 0.037 | 0.78 (0.61, 0.98) | 0.040 |
| Age at natural menopause |  |  |  |  |  |  |
| <47 | 1.32 (1.07, 1.62) | 0.009 | 1.33(1.08, 1.63) | 0.007 | 1.36 (1.10, 1.67) | 0.004 |
| 47-49 | 1.07 (0.91, 1.26) | 0.423 | 1.07 (0.85, 1.34) | 0.572 | 1.08 (0.86, 1.35) | 0.514 |
| 50 (ref) | - | - | - | - | - | - |
| 51-52 | 0.80 (0.68, 0.94) | 0.007 | 0.80 (0.64, 1.00) | 0.050 | 0.80 (0.64, 1.00) | 0.050 |
| 53-54 | 0.76 (0.62, 0.93) | 0.008 | 0.76 (0.59, 0.98) | 0.034 | 0.76 (0.59, 0.98) | 0.034 |
| >54 | 0.93 (0.80, 1.08) | 0.349 | 0.92 (0.75, 1.14) | 0.443 | 0.90 (0.73, 1.12) | 0.340 |
| Hysterectomy vs not | 1.12 (1.01, 1.25) | 0.037 | 1.12 (1.01, 1.25) | 0.037 | 1.12 (1.01, 1.25) | 0.037 |
| Oophorectomy vs not | 1.07 (0.92, 1.24) | 0.381 | 1.07 (0.92, 1.24) | 0.381 | 1.07 (0.92, 1.24) | 0.381 |
| Ever taken oral contraceptive pills | 0.80 (0.72, 0.88) | <0.001 | 0.81 (0.72, 0.90) | <0.001 | 0.77 (0.70, 0.86) | <0.001 |
| Age started oral contraceptive pills (per year) | 1.01 (1.00, 1.03) | 0.188 | 1.01 (1.00, 1.02) | 0.048 | 1.02 (1.00, 1.03) | 0.009 |
| Ever used HRT | 0.99 (0.90, 1.09) | 0.848 | 1.01 (0.91, 1.11) | 0.855 | 0.98 (0.89, 1.09) | 0.709 |
| Age started HRT (per year) | 0.96 (0.95, 0.98) | <0.001 | 0.96 (0.95, 0.98) | <0.001 | 0.96 (0.95, 0.98) | <0.001 |
| Duration of HRT use (per year) | 1.00 (0.98, 1.01) | 0.999 | 1.00 (0.98, 1.01) | 0.999 | 1.00 (0.98, 1.01) | 0.999 |

CI, Confidence Intervals; HR, Hazard Ratio; HRT, Hormone Replacement Therapy; OR, Odds Ratio.

Analyses were adjusted for age, Townsend index, ethnicity, smoking status, systolic blood pressure, body mass index, diabetes, total cholesterol, antihypertensive drugs, lipids lowering drugs.

^a^ These confidence intervals were calculated without the floating absolute risk, to provide comparable results to the competing risk models and the multinomial regression models.
