# Supplementary material for: Reproductive factors and the risk of incident dementia: A cohort study of UK Biobank participants
Source: PLoS Med. 2022 Apr 5;19(4):e1003955. doi: 10.1371/journal.pmed.1003955 (PMC8982865; doi:10.1371/journal.pmed.1003955)
Supplement: S8 Table — Analyses were adjusted for age, Townsend index, ethnicity, smoking status, systolic blood pressure, BMI, diabetes, total cholesterol, antihypertensive drugs, and lipid-lowering drugs. aEarly menarche was defined as age at first menstrual period before the age of 12 years. bEach live birth in women and each child fathered in men. cEarly menopause was defined as the permanent absence of menstrual periods before the age of 47 years. BMI, body mass index; CI, confidence interval; HRT, hormone replacement therapy. (DOCX) [file pmed.1003955.s009.docx]

**S8 Table: Multiple-adjusted Hazard Ratios (95% confidence intervals) for the risk of dementia associated with reproductive factors stratified by Townsend fifths.**

| **Reproductive factor** |  | **Townsend fifths** | | | | | |
| --- | --- | --- | --- | --- | --- | --- | --- |
|  | **First**  **(Least disadvantaged)**  **(<-2.938)** | | **Second**  **(≥-2.938, <1.531)** | **Third**  **(≥-1.531, <0.170)** | **Fourth**  **(≥0.170, 2.448)** | **Fifth**  **(Most disadvantaged)**  **(≥2.448)** | **P for heterogeneity across Townsend fifths** |
| Early menarche ^a^ vs. not | 1.23 (1.00, 1.52) | | 0.96 (0.72, 1.28) | 1.40 (1.05, 1.87) | 1.06 (0.76, 1.48) | 1.07 (0.81, 1.41) | 0.382 |
| Age at first live birth per year | 1.00 (0.98, 1.02) | | 0.99 (0.97, 1.02) | 0.98 (0.95, 1.01) | 0.99 (0.96, 1.03) | 0.96 (0.93, 0.98) | 0.184 |
| Each child ^b^ |  | |  |  |  |  |  |
| Women | 0.90 (0.83, 0.97) | | 0.96 (0.87, 1.06) | 0.94 (0.85, 1.05) | 1.06 (0.96, 1.17) | 1.06 (0.98, 1.13) | 0.015 |
| Men | 1.00 (0.93, 1.07) | | 1.00 (0.92, 1.10) | 1.05 (0.96, 1.14) | 1.03 (0.95, 1.12) | 1.04 (1.00, 1.09) | 0.831 |
| Stillbirth vs. not | 1.47 (0.96, 2.26) | | 1.03 (0.55, 1.95) | 0.74 (0.33, 1.66) | 1.16 (0.59, 2.27) | 1.16 (0.71, 1.87) | 0.650 |
| Miscarriage vs. not | 0.90 (0.72, 1.12) | | 0.92 (0.68, 1.23) | 0.82 (0.58, 1.15) | 0.93 (0.66, 1.33) | 1.11 (0.84, 1.46) | 0.704 |
| Abortion vs. not | 0.87 (0.62, 1.21) | | 0.66 (0.41, 1.06) | 1.03 (0.68, 1.55) | 0.99 (0.65, 1.51) | 0.82 (0.58, 1.16) | 0.659 |
| Early menopause ^c^ vs. not | 1.26 (0.95, 1.66) | | 1.28 (0.88, 1.86) | 1.36 (0.92, 2.02) | 1.90 (1.27, 2.85) | 1.78 (1.28, 2.48) | 0.322 |
| Hysterectomy vs. not | 0.94 (0.77, 1.14) | | 0.97 (0.75, 1.25) | 1.23 (0.94, 1.61) | 1.40 (1.05, 1.87) | 1.33 (1.05, 1.68) | 0.059 |
| Oophorectomy vs. not | 0.79 (0.58, 1.06) | | 0.75 (0.50, 1.11) | 1.31 (0.92, 1.87) | 1.83 (1.28, 2.62) | 1.23 (0.89, 1.69) | 0.002 |
| Oral contraceptive pill use vs. not | 1.01 (0.84, 1.22) | | 0.75 (0.59, 0.95) | 0.79 (0.61, 1.03) | 0.65 (0.49, 0.87) | 0.69 (0.55, 0.87) | 0.042 |
| HRT use vs. not | 0.93 (0.79, 1.11) | | 0.90 (0.72, 1.12) | 0.98 (0.77, 1.26) | 1.18 (0.89, 1.56) | 1.09 (0.87, 1.37) | 0.488 |

HRT, Hormone Replacement Therapy.

Analyses were adjusted for age, Townsend index, ethnicity, smoking status, systolic blood pressure, body mass index, diabetes, total cholesterol, antihypertensive drugs, lipids lowering drugs.

^a^ Early menarche was defined as age at first menstrual period before the age of 12 years.

^b^ Each livebirth in women and each child fathered in men.

^c^ Early menopause was defined as the permanent absence of menstrual periods before the age of 47 years.
