# Supplementary material for: Reproductive factors and the risk of incident dementia: A cohort study of UK Biobank participants
Source: PLoS Med. 2022 Apr 5;19(4):e1003955. doi: 10.1371/journal.pmed.1003955 (PMC8982865; doi:10.1371/journal.pmed.1003955)
Supplement: S9 Table — Analyses were adjusted for age, Townsend index, ethnicity, smoking status, systolic blood pressure, BMI, diabetes, total cholesterol, antihypertensive drugs, and lipid-lowering drugs. aWeighted HR are weighted according to equal weights of each stratified HR, to represent that the national Townsend fifths contain equal number of people. BMI, body mass index; CI, confidence interval; HR, hazard ratio; HRT, hormone replacement therapy. (DOCX) [file pmed.1003955.s010.docx]

| **Reproductive factor** | **Townsend-weighted HR (95% CI) ^a^** | **P-value** |
| --- | --- | --- |
| Age at menarche |  |  |
| <12 | 1.20 (0.94, 1.54) | 0.148 |
| 12 | 1.09 (0.84, 1.43) | 0.536 |
| 13 (ref) | 1.00 (0.78, 1.29) | - |
| 14 | 0.96 (0.73, 1.26) | 0.840 |
| >14 | 1.19 (0.92, 1.54) | 0.187 |
| Number of live births |  |  |
| 0 | 1.15 (0.86, 1.53) | 0.347 |
| 1 | 0.96 (0.73, 1.43) | 0.825 |
| 2 (ref) | 1.00 (0.84, 1.20) | - |
| 3 | 1.05 (0.82, 1.33) | 0.706 |
| 4 or more | 1.10 (0.77, 1.58) | 0.616 |
| Age at first live birth |  |  |
| <21 | 1.38 (1.03, 1.84) | 0.030 |
| 21-22 | 1.25 (0.94, 1.67) | 0.128 |
| 23-24 | 1.33 (1.01, 1.76) | 0.044 |
| 25-26 (ref) | 1.00 (0.70, 1.42) | - |
| 27-29 | 1.21 (0.89, 1.66) | 0.233 |
| >29 | 1.07 (0.74, 1.53) | 0.728 |
| Number of miscarriages |  |  |
| 0 (ref) | 1.00 (0.87, 1.15) | - |
| 1 | 0.89 (0.64, 1.22) | 0.489 |
| 2 or more | 1.03 (0.65, 1.65) | 0.909 |
| Number of stillbirths |  |  |
| 0 (ref) | 1.00 (0.88, 1.14) | - |
| 1 | 1.07 (0.56, 2.02) | 0.847 |
| 2 or more | 1.49 (0.32, 7.09) | 0.626 |
| Number of abortions |  |  |
| 0 (ref) | 1.00 (0.86, 1.16) | - |
| 1 | 0.98 (0.67, 1.43) | 0.924 |
| 2 or more | 0.42 (0.12, 1.48) | 0.177 |
| Reproductive years |  |  |
| <33 (ref) | 1.00 (0.70, 1.43) | - |
| 33-35 | 0.94 (0.65, 1.36) | 0.755 |
| 36-37 | 0.77 (0.53, 1.13) | 0.177 |
| 38-39 | 0.68 (0.46, 0.99) | 0.048 |
| 40-42 | 0.64 (0.44, 0.93) | 0.019 |
| >42 | 0.75 (0.48, 1.15) | 0.198 |
| Age at natural menopause |  |  |
| <47 | 1.32 (0.96, 1.79) | 0.080 |
| 47-49 | 1.02 (0.69, 1.51) | 0.928 |
| 50 (ref) | 1.00 (0.69, 1.43) | - |
| 51-52 | 0.76 (0.52, 1.13) | 0.166 |
| 53-54 | 0.70 (0.43, 1.16) | 0.159 |
| >54 | 0.89 (0.62, 1.28) | 0.540 |
| Hysterectomy vs not | 1.16 (0.90, 1.48) | 0.245 |
| Oophorectomy vs not | 1.12 (0.79, 1.58) | 0.532 |
| Ever taken oral contraceptive pills | 0.77 (0.61, 0.98) | 0.030 |
| Age started oral contraceptive pills (per year) | 1.01 (0.97, 1.04) | 0.588 |
| Ever used HRT | 1.01 (0.81, 1.27) | 0.937 |
| Age started HRT (per year) | 0.96 (0.93, 0.99) | 0.010 |
| Duration of HRT use (per year) | 0.99 (0.96, 1.03) | 0.588 |

**S9 Table: Townsend-weighted multiple-adjusted hazard ratios (95% confidence intervals) for the risk of dementia associated with reproductive factors.**

HRT, Hormone Replacement Therapy.

Analyses were adjusted for age, Townsend index, ethnicity, smoking status, systolic blood pressure, body mass index, diabetes, total cholesterol, antihypertensive drugs, lipids lowering drugs.

^a^ Weighted HR are weighted according to equal weights of each stratified HR, to represent that the national Townsend fifths contain equal number of people.
