# Supplementary material for: Reproductive factors and the risk of incident dementia: A cohort study of UK Biobank participants
Source: PLoS Med. 2022 Apr 5;19(4):e1003955. doi: 10.1371/journal.pmed.1003955 (PMC8982865; doi:10.1371/journal.pmed.1003955)
Supplement: S10 Table — (A) A consideration of pregnancy related factors: number of live births, stillbirths, miscarriages, and abortions, although there were no significant change in the model coefficients in this model compared with the individual associations. Analyses were adjusted for age, Townsend index, ethnicity, smoking status, systolic blood pressure, BMI, diabetes, total cholesterol, antihypertensive drugs, and lipid-lowering drugs. (B) A consideration of factors throughout the life span including age at menarche, parous versus not, hysterectomy and or oophorectomy, HRT use, and contraceptive pill use. Analyses were adjusted for age, Townsend index, ethnicity, smoking status, systolic blood pressure, BMI, diabetes, total cholesterol, antihypertensive drugs, and lipid-lowering drugs. BMI, body mass index; CI, confidence interval; HRT, hormone replacement therapy. (DOCX) [file pmed.1003955.s011.docx]

**S10 Table: Multiple-adjusted hazard ratios (95% confidence intervals) for comparing individual and combined associations between reproductive factors and dementia.**

| **Model 1** | **Individual associations**  **HR (95% CI)** | **p-value** | **Combined associations**  **HR (95% CI)** | **p-value** |
| --- | --- | --- | --- | --- |
| Per live birth | 0.98 (0.95, 1.02) | 0.269 | 1.02 (0.97, 1.07) | 0.437 |
| Per miscarriage | 1.01 (0.94, 1.08) | 0.791 | 1.01 (0.94, 1.08) | 0.791 |
| Per stillbirth | 1.10 (0.92, 1.31) | 0.294 | 1.09 (0.90, 1.31) | 0.375 |
| Per abortion | 0.82 (0.71, 0.94) | 0.006 | 0.82 (0.71, 0.94) | 0.006 |

**A.** A consideration of pregnancy related factors: number of live births, stillbirths, miscarriages, and abortions, though there were no significant change in the model coefficients in this model compared with the individual associations.

Analyses were adjusted for age, Townsend index, ethnicity, smoking status, systolic blood pressure, body mass index, diabetes, total cholesterol, antihypertensive drugs, lipids lowering drugs.

**B.** A consideration of factors throughout the lifespan including age at menarche, parous vs not, hysterectomy and or oophorectomy, HRT use, contraceptive pill use.

| **Model 2** | **Individual associations**  **HR (95%CI)** | **p-value** | **Combined associations**  **HR (95%CI)** | **p-value** |
| --- | --- | --- | --- | --- |
| Age at menarche |  |  |  |  |
| <12 | 1.20 (1.08, 1.34) | <0.001 | 1.19 (1.06, 1.32) | 0.002 |
| 12 | 1.07 (0.95, 1.20) | 0.259 | 1.08 (0.96, 1.22) | 0.210 |
| 13 (ref) | 1.00 (0.90, 1.11) | - | 1.00 (0.90, 1.12) | - |
| 14 | 0.97 (0.87, 1.09) | 0.609 | 0.98 (0.87, 1.10) | 0.749 |
| >14 | 1.19 (1.07, 1.34) | 0.002 | 1.18 (1.05, 1.32) | 0.005 |
|  |  |  |  |  |
| Parous vs not | 0.88 (0.77, 1.01) | 0.064 | 0.92 (0.80, 1.07) | 0.264 |
|  |  |  |  |  |
| No hysterectomy or oophorectomy (ref) | 1.00 (0.96, 1.06) | - | 1.00 (0.94, 1.07) | - |
| Hysterectomy without oophorectomy | 1.13 (1.00, 1.28) | 0.052 | 1.13 (1.00, 1.29) | 0.060 |
| Hysterectomy with oophorectomy | 1.09 (0.95, 1.26) | 0.234 | 1.11 (0.96, 1.29) | 0.167 |
|  |  |  |  |  |
| Ever used HRT | 0.99 (0.90, 1.09) | 0.848 | 1.00 (0.90, 1.11) | 1.000 |
| Ever taken oral contraceptive pills | 0.80 (0.72, 0.88) | <0.001 | 0.82 (0.73, 0.92) | <0.001 |

HRT, Hormone Replacement Therapy.

Analyses were adjusted for age, Townsend index, ethnicity, smoking status, systolic blood pressure, body mass index, diabetes, total cholesterol, antihypertensive drugs, lipids lowering drugs.
