# Supplementary material for: Reproductive factors and the risk of incident dementia: A cohort study of UK Biobank participants
Source: PLoS Med. 2022 Apr 5;19(4):e1003955. doi: 10.1371/journal.pmed.1003955 (PMC8982865; doi:10.1371/journal.pmed.1003955)
Supplement: S11 Table — Analyses were adjusted for age, Townsend index, ethnicity, smoking status, systolic blood pressure, BMI, diabetes, total cholesterol, antihypertensive drugs, and lipid-lowering drugs. BMI, body mass index; CI, confidence interval; HR, hazard ratio; HRT, hormone replacement therapy; MICE, Multivariate Imputation by Chained Equations. (DOCX) [file pmed.1003955.s012.docx]

**S11 Table: Multiple-adjusted hazard ratios for the risk of dementia associated with reproductive factors in women, after imputed for missing data using Multivariate Imputation via Chained Equations (MICE), compared with complete case analyses.**

| **Reproductive factor** | **Imputed with MICE** | | **Complete case** | |
| --- | --- | --- | --- | --- |
|  | **Multiple-adjusted HR**  **(95% CI)** | **P-value** | **Multiple-adjusted HR**  **(95% CI)** | **P-value** |
| Age at menarche |  |  |  |  |
| <12 | 1.16 (1.05, 1.28) | 0.004 | 1.20 (1.08, 1.34) | 0.016 |
| 12 | 1.10 (0.99, 1.22) | 0.074 | 1.07 (0.95, 1.20) | 0.389 |
| 13 (ref) | 1.00 (0.91, 1.10) | - | 1.00 (0.90, 1.11) | - |
| 14 | 0.99 (0.89, 1.10) | 0.863 | 0.97 (0.87, 1.09) | 0.718 |
| >14 | 1.16 (1.04, 1.28) | 0.006 | 1.19 (1.07, 1.34) | 0.024 |
| Ever been pregnant | 0.86 (0.75, 0.99) | 0.036 | 0.85 (0.74, 0.98) | 0.026 |
| Number of live births |  |  |  |  |
| 0 | 1.11 (0.99, 1.26) | 0.079 | 1.18 (1.04, 1.33) | 0.027 |
| 1 | 1.01 (0.90, 1.14) | 0.855 | 1.09 (0.95, 1.25) | 0.276 |
| 2 (ref) | 1.00 (0.92, 1.09) | - | 1.00 (0.93, 1.08) | - |
| 3 | 1.04 (0.95, 1.15) | 0.406 | 1.03 (0.93, 1.15) | 0.641 |
| 4 or more | 1.12 (0.98, 1.29) | 0.106 | 1.14 (0.98, 1.33) | 0.132 |
| Parous vs not | 0.88 (0.78, 1.01) | 0.071 | 0.88 (0.77, 1.01) | 0.061 |
| Per live birth | 0.99 (0.96, 1.03) | 0.689 | 0.98 (0.95, 1.02) | 0.430 |
| Age at first live birth |  |  |  |  |
| <21 | 1.44 (1.28, 1.62) | <0.001 | 1.43 (1.26, 1.62) | <0.001 |
| 21-22 | 1.23 (1.08, 1.39) | 0.001 | 1.23 (1.08, 1.40) | 0.034 |
| 23-24 | 1.30 (1.15, 1.46) | <0.001 | 1.26 (1.12, 1.42) | 0.015 |
| 25-26 (ref) | 1.00 (0.87, 1.15) | - | 1.00 (0.87, 1.15) | - |
| 27-29 | 1.04 (0.91, 1.20) | 0.585 | 1.16 (1.02, 1.32) | 0.129 |
| >29 | 1.04 (0.87, 1.23) | 0.704 | 1.11 (0.96, 1.29) | 0.296 |
| Per additional year of age at first live birth | 0.97 (0.96, 0.98) | <0.001 | 0.98 (0.97, 1.00) | 0.006 |
| Number of miscarriages |  |  |  |  |
| 0 (ref) | 1.00 (0.95, 1.06) | - | 1.00 (0.94, 1.06) | - |
| 1 | 0.94 (0.83, 1.07) | 0.353 | 0.90 (0.78, 1.03) | 0.147 |
| 2 or more | 1.03 (0.85, 1.24) | 0.791 | 1.02 (0.83, 1.25) | 0.847 |
| Miscarriage vs not | 0.97 (0.86, 1.09) | 0.571 | 0.93 (0.82, 1.06) | 0.274 |
| Per miscarriage | 1.02 (0.96, 1.08) | 0.605 | 1.01 (0.94, 1.08) | 0.816 |
| Number of stillbirths |  |  |  |  |
| 0 (ref) | 1.00 (0.95, 1.06) | - | 1.00 (0.94, 1.06) | - |
| 1 | 1.17 (0.92, 1.49) | 0.196 | 1.15 (0.88, 1.49) | 0.312 |
| 2 or more | 1.39 (0.79, 2.45) | 0.257 | 1.27 (0.66, 2.45) | 0.472 |
| Stillbirth vs not | 1.20 (0.96, 1.51) | 0.116 | 1.16 (0.91, 1.49) | 0.234 |
| Per stillbirth | 1.11 (0.95, 1.31) | 0.185 | 1.10 (0.92, 1.31) | 0.319 |
| Number of abortions |  |  |  |  |
| 0 (ref) | 1.00 (0.94, 1.06) | - | 1.00 (0.94, 1.07) | - |
| 1 | 0.96 (0.82, 1.12) | 0.637 | 0.97 (0.82, 1.14) | 0.735 |
| 2 or more | 0.41 (0.24, 0.69) | <0.001 | 0.34 (0.18, 0.64) | <0.001 |
| Abortion vs not | 0.87 (0.74, 1.02) | 0.096 | 0.87 (0.73, 1.03) | 0.101 |
| Per abortion | 0.83 (0.73, 0.95) | 0.007 | 0.82 (0.71, 0.94) | 0.006 |
| Reproductive years |  |  |  |  |
| <33 (ref) | 1.00 (0.89, 1.13) | - | 1.00 (0.86, 1.17) | - |
| 33-35 | 0.93 (0.82, 1.05) | 0.262 | 0.98 (0.84, 1.15) | 0.878 |
| 36-37 | 0.79 (0.70, 0.89) | <0.001 | 0.78 (0.67, 0.92) | 0.031 |
| 38-39 | 0.67 (0.59, 0.76) | <0.001 | 0.67 (0.57, 0.79) | <0.001 |
| 40-42 | 0.69 (0.61, 0.78) | <0.001 | 0.68 (0.58, 0.79) | <0.001 |
| >42 | 0.68 (0.58, 0.78) | <0.001 | 0.80 (0.67, 0.95) | 0.056 |
| Age at natural menopause |  |  |  |  |
| <47 | 1.29 (1.15, 1.44) | <0.001 | 1.32 (1.15, 1.51) | 0.008 |
| 47-49 | 1.16 (1.02, 1.32) | 0.023 | 1.07 (0.91, 1.26) | 0.573 |
| 50 (ref) | 1.00 (0.88, 1.13) | - | 1.00 (0.86, 1.17) | - |
| 51-52 | 0.94 (0.83, 1.06) | 0.287 | 0.80 (0.68, 0.94) | 0.048 |
| 53-54 | 0.89 (0.77, 1.04) | 0.136 | 0.76 (0.62, 0.93) | 0.035 |
| >54 | 0.88 (0.78, 1.00) | 0.046 | 0.93 (0.80, 1.08) | 0.496 |
| Hysterectomy vs not | 1.11 (1.00, 1.22) | 0.053 | 1.12 (1.01, 1.25) | 0.039 |
| Oophorectomy vs not | 1.08 (0.94, 1.25) | 0.263 | 1.07 (0.92, 1.24) | 0.413 |
| Ever taken oral contraceptive pills | 0.79 (0.72, 0.88) | <0.001 | 0.80 (0.72, 0.88) | <0.001 |
| Age started oral contraceptive pills (per year) | 1.02 (1.00, 1.03) | 0.009 | 1.01 (1.00, 1.03) | 0.143 |
| Ever used HRT | 0.96 (0.87, 1.05) | 0.367 | 0.99 (0.90, 1.09) | 0.828 |
| Age started HRT (per year) | 0.96 (0.95, 0.98) | <0.001 | 0.96 (0.95, 0.98) | <0.001 |
| Duration of HRT use (per year) | 1.00 (0.98, 1.01) | 0.521 | 1.00 (0.98, 1.01) | 0.526 |

CI, Confidence Intervals; HR, Hazard Ratio; HRT, Hormone Replacement Therapy.

Analyses were adjusted for age, Townsend index, ethnicity, smoking status, systolic blood pressure, body mass index, diabetes, total cholesterol, antihypertensive drugs, lipids lowering drugs.
