# Supplementary material for: Reproductive factors and the risk of incident dementia: A cohort study of UK Biobank participants
Source: PLoS Med. 2022 Apr 5;19(4):e1003955. doi: 10.1371/journal.pmed.1003955 (PMC8982865; doi:10.1371/journal.pmed.1003955)
Supplement: Attachment — Submitted filename: gong.pdf [file pmed.1003955.s013.pdf]

This paper uses data from the UK Biobank to link reproductive factors and incident dementia.

I was asked for a statistical report and I interpret that to include all aspects of the design and conduct of the study.

Points of detail

Page 2 Something seems to have gone missing in the sentence starting 'There was evidence'. In any event it needs the effect size and confidence interval.

Page 3 I feel that the authors should later tell us how they square the higher prevalence in women with the lower incidence rates which they found.

Page 6 The finding of 263 prevalent cases is part of the results although the decision to exclude them is indeed part of the methods.

Figure 1 I am not sure which distribution was truncated at its extremes. Is this for the kernel plots? Incidentally in the version I received they are just a faint smudge and I almost missed them. Some of the age variables seem to show quite pronounced digit preference which perhaps limits their value.

Has the composition of hormonal contraception changed over time and if so does that matter for inferences?

Points of more substance

Missing data

On page 7 we learn that the authors used complete data in their models. As far as I can see the amount of this is not reported, apologies if I missed it. However I think there are some clues in Table 1. If we look at miscarriages, for example, we find that the values given do not add up to 100% leaving approximately 16.8% unaccounted for. Other variables have similar rates of missing. Even if this is the same women each time it raises serious questions about the wisdom of using complete cases. Some attempt at imputation is called for here. We also need to see the number of missing for all variables as if 828 women cannot or will not tell us how many children they have had it does suggest many other variables have even more missing.

Representativeness of UK Biobank

As the authors mention on page 23 the UK Biobank is known not to represent the UK population. In a different field (alcohol studies) Stamatakis et al. (2021) have shown that adjusting for the lack of representativeness made a profound difference. I think the authors would be well advised to consider similar supplementary analyses to reassure us. There must be national statistics on some of these variables. For instance the NHS website `https://www.nhs.uk/conditions/stillbirth/` assures us that approximately 1 in 200 pregnancies (0.5%) ends in a still birth. Assuming they are independent this suggests that women with two pregnancies would have approximately a 1% chance of at least one stillbirth whereas about 2.6% of the women here reported at least one.

Form of relationship

Having shown us that the relationship between some variables is U–shaped the authors provide extensive coverage in the results and discussion of analysis per year which supposes a linear relationship. This is clearly inconsistent. I know it is difficult to explain and interpret the interaction between a covariate and a quadratic term but the linear results cannot stand.

Summary

Some fairly major issues to sort out.

Michael Dewey

References

E Stamatakis, K B Owen, L Shepherd, B Drayton, M Hamer, and A E Bauman. Is cohort representativeness *passé*? Poststratified associations of lifestyle risk factors with mortality in the UK Biobank. *Epidemiology*, 32: 179–188, 2021.
