# Supplementary material for: Reproductive factors and the risk of incident dementia: A cohort study of UK Biobank participants
Source: PLoS Med. 2022 Apr 5;19(4):e1003955. doi: 10.1371/journal.pmed.1003955 (PMC8982865; doi:10.1371/journal.pmed.1003955)
Supplement: Attachment — Submitted filename: gong2.pdf [file pmed.1003955.s015.pdf]

This paper uses data from the UK Biobank to link reproductive factors and incident dementia.

I was asked for a statistical report and I interpret that to include all aspects of the design and conduct of the study. When I am asked to look at a re–submission which I had already reviewed on a previous submission I restrict myself on principle to commenting on how the authors have responded to my original comments. I do not review the article again from scratch unless it has been so changed as to amount effectively to a new submission.

I said:

> *The finding of 263 prevalent cases is part of the results although the decision to exclude them is indeed part of the methods.*

Perhaps the authors misunderstood my point here. I agree that deciding to remove prevalent cases is part of the methods but the actual number involved was something that was only discovered during analysis and so is part of the results. I agree this is rather pedantic but it is still better to keep results all in the same place.

I said:

> *On page 7 we learn that the authors used complete data in their models. As far as I can see the amount of this is not reported, apologies if I missed it. However I think there are some clues in Table 1. If we look at miscarriages, for example, we find that the values given do not add up to 100% leaving approximately 16.8% unaccounted for. Other variables have similar rates of missing. Even if this is the same women each time it raises serious questions about the wisdom of using complete cases. Some attempt at imputation is called for here. We also need to see the number of missing for all variables as if 828 women cannot or will not tell us how many children they have had it does suggest many other variables have even more missing.*

The authors have reported more detail about the numbers missing but have ignored my point about using some form of multiple imputation. I was expecting then either to provide the scientific reasons for rejecting this or to do it.

I said:

> *Having shown us that the relationship between some variables is U–shaped the authors provide extensive coverage in the results and discussion of analysis per year which supposes a linear relation-*

> *ship. This is clearly inconsistent. I know it is difficult to explain and interpret the interaction between a covariate and a quadratic term but the linear results cannot stand.*

There are still places where following the finding of a U–shaped relationship the authors then interpret it as linear. I did not do an exhaustive search but, for instance, on page 21 we read "Compared with those who had two children, the associations between the number of children and dementia were similar for women and men and were U-shaped (Figure 2). [. . .] each additional child was associated with lower dementia risk in women [. . .]. If the relationship is not linear then it is misleading to speak of the association of each additional child as non–linearity implies that is not constant across the range.

Summary

Still some issues to sort out.

Michael Dewey
