# Supplementary material for: Reproductive factors and the risk of incident dementia: A cohort study of UK Biobank participants
Source: PLoS Med. 2022 Apr 5;19(4):e1003955. doi: 10.1371/journal.pmed.1003955 (PMC8982865; doi:10.1371/journal.pmed.1003955)
Supplement: Attachment — Submitted filename: Responses to Editor and referees_PMEDICINE-D-21-01592R1.docx [file pmed.1003955.s014.docx]

**Responses to Editor and referees:**

**Gong et al.** **Reproductive factors and the risk of incident dementia: a prospective cohort study of UK Biobank participants (PMEDICINE-D-21-01592R1)**

We thank the Editor and referees for taking their time to review our manuscript, and for providing us with the opportunity to revise and improve it. We have addressed the comments point by point. Changes made in the manuscript are within quotation marks and are in italics. We provide a marked-up version of the manuscript, highlighting all changes and new material included in the manuscript.

An important change since our original submission is that we have updated all analyses with additional follow-up data in the UK Biobank, to provide the most reliable results possible from the study, with longer follow-up time. The UK Biobank updates its follow-up data periodically, and the participants are free to withdraw from the study at any given time. Since our initial submission we now have provided updated data accounting for withdrawals with follow-up to 30 November 2020.

Further to this important change, we have conducted additional analyses, taking comments from the editor and all four reviewers into account. Our study has been improved by analysing the timing of hormone use in relation to menopause, as well as post-hoc sensitivity analyses included death as a competing risk in estimating the associations between reproductive factors and dementia risk, using Fine and Gray competing risk models, and multinomial regression models. Combinations of reproductive factors, rather than examined in isolation were evaluated; Analyses were additionally reweighted according to the social structure of the population in general, with these results included in the supplementary materials.

**Requests from the editors:**

**1. Please address the following comment from the Academic Editor: The paper gives a detailed look at this issue, but there are a couple of major areas of concern – one is the challenge of interpretation of the results given the low response rate for UK Biobank and the likelihood that those who took part from lower SES seem unlikely to fully represent these populations. The findings suggest residual confounding with these factors.**

Thank you for this important comment. The UK Biobank is, indeed, not representative of the UK general population regarding sociodemographic, physical, lifestyle and health-related characteristics. But this does not necessarily mean that it is not suitable for examining associations between exposure measures with health outcomes (Woodward, 2014). Indeed, many authors (e.g., Fry et al, 2016; Batty et al, 2020) – including our own work (e.g. Millett et al, 2018; and Peters et al, 2020) - have shown that such results from the UK Biobank are similar, but with more precision due to the large sample size - to those found from bespoke cohort studies. We agree that residual confounding may be present and have now acknowledged that in our limitations section on page 28, lines 447-451, but that is true for any epidemiological study, whilst clearly a randomised controlled trial would not be possible in our area of research. The key issue is whether the social imbalance you point out has biased our findings. We have addressed this question by reweighting our analyses according to the social structure of the population in general (S6 Table), based on Reviewer 4’s suggestion (Stamatakis et al 2021).

These are included in the methods, see page 10, lines 202-206:

*“**In addition, the results were weighted according to the social structure of the population in general: Weighted HR were calculated according to equal weights of each stratified HR by fifths of Townend Score, since the national Townsend fifths, by definition, contain an equal number of people.”*

And in results, page 24, lines 347-350:

*“... After weighting the estimates by Townsend fifths according to the national distribution, the pooled estimates were similar to those in the main analysis but with wider confidence intervals, caused by giving relatively higher weights to the fifths with the least precise estimates (S6 Table).”*

This was also included in the limitations section on page 30, lines 466-469.

*“**After the results were weighted by Townsend fifths, there was extra imprecision in the weighted HR estimates for some exposures, which occurred where there is considerable difference in estimates (HR and standard errors) across the Townsend fifths; however, this may be a result of small numbers in some exposure categories.”*

| **Risk factors** | **Townsend-weighted HR (95% CI) ^a^** |
| --- | --- |
| Age at menarche |  |
| <12 | 1.20 (0.94, 1.54) |
| 12 | 1.09 (0.84, 1.43) |
| 13 (ref) | 1.00 (0.78, 1.29) |
| 14 | 0.96 (0.73, 1.26) |
| >14 | 1.19 (0.92, 1.54) |
| Number of live births |  |
| 0 | 1.15 (0.86, 1.53) |
| 1 | 0.96 (0.73, 1.43) |
| 2 (ref) | 1.00 (0.84, 1.20) |
| 3 | 1.05 (0.82, 1.33) |
| 4 or more | 1.10 (0.77, 1.58) |
| Age at first live birth |  |
| <21 | 1.38 (1.03, 1.84) |
| 21-22 | 1.25 (0.94, 1.67) |
| 23-24 | 1.33 (1.01, 1.76) |
| 25-26 (ref) | 1.00 (0.70, 1.42) |
| 27-29 | 1.21 (0.89, 1.66) |
| >29 | 1.07 (0.74, 1.53) |
| Number of miscarriages |  |
| 0 (ref) | 1.00 (0.87, 1.15) |
| 1 | 0.89 (0.64, 1.22) |
| 2 or more | 1.03 (0.65, 1.65) |
| Number of stillbirths |  |
| 0 (ref) | 1.00 (0.88, 1.14) |
| 1 | 1.07 (0.56, 2.02) |
| 2 or more | 1.49 (0.32, 7.09) |
| Number of abortions |  |
| 0 (ref) | 1.00 (0.86, 1.16) |
| 1 | 0.98 (0.67, 1.43) |
| 2 or more | 0.42 (0.12, 1.48) |
| Reproductive years |  |
| <33 (ref) | 1.00 (0.70, 1.43) |
| 33-35 | 0.94 (0.65, 1.36) |
| 36-37 | 0.77 (0.53, 1.13) |
| 38-39 | 0.68 (0.46, 0.99) |
| 40-42 | 0.64 (0.44, 0.93) |
| >42 | 0.75 (0.48, 1.15) |
| Age at natural menopause |  |
| <47 | 1.32 (0.96, 1.79) |
| 47-49 | 1.02 (0.69, 1.51) |
| 50 (ref) | 1.00 (0.69, 1.43) |
| 51-52 | 0.76 (0.52, 1.13) |
| 53-54 | 0.70 (0.43, 1.16) |
| >54 | 0.89 (0.62, 1.28) |
| Hysterectomy vs not | 1.16 (0.90, 1.48) |
| Oophorectomy vs not | 1.12 (0.79, 1.58) |
| Ever taken oral contraceptive pills | 0.77 (0.61, 0.98) |
| Age started oral contraceptive pills (per year) | 1.01 (0.97, 1.04) |
| Ever used HRT | 1.01 (0.81, 1.27) |
| Age started HRT (per year) | 0.96 (0.93, 0.99) |
| Duration of HRT use (per year) | 0.99 (0.96, 1.03) |

**S6 Table: Townsend-weighted multiple-adjusted hazard ratios (95% confidence intervals) for the risk of dementia associated with reproductive factors.**

HRT, Hormone Replacement Therapy.

Analyses were adjusted for age, Townsend index, ethnicity, smoking status, systolic blood pressure, body mass index, diabetes, total cholesterol, antihypertensive drugs, lipids lowering drugs.

^a^ Weighted HR are weighted according to equal weights of each stratified HR, to represent that the national Townsend fifths contain equal number of people.

Reference:

- Woodward M. Epidemiology: Study Design and Data Analysis. 3rd edn. Boca Raton: CRC Press, 2014. ISBN-13: 978-1-4398-3970-6.
- Fry, A., Littlejohns, T. J., Sudlow, C., Doherty, N., & Allen, N. E. (2016). OP41 The representativeness of the UK Biobank cohort on a range of sociodemographic, physical, lifestyle and health-related characteristics.
- Batty, G. D., Gale, C. R., Kivimäki, M., Deary, I. J., & Bell, S. (2020). Comparison of risk factor associations in UK Biobank against representative, general population based studies with conventional response rates: prospective cohort study and individual participant meta-analysis. bmj, 368.
- Millett, E. R., Peters, S. A., & Woodward, M. (2018). Sex differences in risk factors for myocardial infarction: cohort study of UK Biobank participants. bmj, 363.
- Peters, S. A., Carcel, C., Millett, E. R., & Woodward, M. (2020). Sex differences in the association between major risk factors and the risk of stroke in the UK Biobank cohort study. Neurology, 95(20), e2715-e2726.
- Stamatakis, E., Owen, K. B., Shepherd, L., Drayton, B., Hamer, M., & Bauman, A. E. (2021). Is Cohort Representativeness Passé? Poststratified associations of lifestyle risk factors with mortality in the UK Biobank. Epidemiology (Cambridge, Mass.), 32(2), 179.

**2. Title: Please revise your title according to PLOS Medicine’s style. Your title must be nondeclarative and not a question. It should begin with main concept if possible. “Effect of” should be used only if causality can be inferred, i.e., for an RCT. Please place the study design (“A randomized controlled trial,” “A retrospective study,” “A modelling study,” etc.) in the subtitle (ie, after a colon).**

Thank you. We have now revised the title according to PLOS Medicine’s style. The new title of the study is *“Reproductive factors and the risk of incident dementia: a prospective cohort study of UK Biobank participants”*.

**3. Abstract Background: Provide the context of why the study is important. The final sentence should clearly state the study question.**

Thank you for the suggestion, we have now revised the abstract background to highlight the gaps in evidence and the broad context. Please see page 2, lines 26-27:

*“**Background: Women’s reproductive factors have been associated with the risk of dementia; however, these findings remain equivocal.”*

**4. Abstract: Methods and Findings: Please quantify the main results presented in the abstract with both 95% CIs and p values.**

Thank you, we have presented the hazard ratios and accompanying 95% CIs as well as the p values in the main results.

**5. Abstract: Methods and Findings: Please mention the important dependent variables that are adjusted for in the analyses.**

We have now included the important dependent variables that are adjusted for in the analyses in the methods and findings section of the abstract. Please see page 2, lines 33-36:

*“**Multiple adjusted models included age at study entry, SES, ethnicity, smoking status, systolic blood pressure, BMI, history of diabetes mellitus, total cholesterol, antihypertensive drugs, and lipid-lowering drugs.”*

**6. Abstract: Methods and Findings: In the last sentence of the Abstract Methods and Findings section, please describe the main limitation(s) of the study's methodology.**

We have included the following sentence stating the main limitations of the study’s methodology, please see page 3, lines 48-50:

*“**The main limitations of the study include potential residual confounding, and self-reported measures of reproductive factors.”*

**7. Abstract: Conclusions: Please address the study implications without overreaching what can be concluded from the data; the phrase “In this study, we observed …” may be useful.**

Thank you for this useful suggestion, we have revised the conclusion in the abstract to include the following, please see page 3, lines 51-53:

*“**Conclusions: In this study, we observed that the reproductive events related to shorter cumulative endogenous estrogen exposure in women were associated with higher dementia risk, and there was a similar association between the number of children and dementia risk in women and men.”*

**8. Author Summary: At this stage, we ask that you include a short, non-technical Author Summary of your research to make findings accessible to a wide audience that includes both scientists and non-scientists. The Author Summary should immediately follow the Abstract in your revised manuscript. This text is subject to editorial change and should be distinct from the scientific abstract. Please see our author guidelines for more information:** [**https://journals.plos.org/plosmedicine/s/revising-your-manuscript#loc-author-summary**](https://journals.plos.org/plosmedicine/s/revising-your-manuscript#loc-author-summary)

Thank you. The Author Summary is provided below, and on page 4, lines 54-75 in the main manuscript document:

*“**Why was this study done?*

- *Dementia rates are increasing around the world, with some studies reporting a higher incidence in women than men.*
- *Evidence on the associations between reproductive factors and the risk of dementia remains equivocal.*
- *Hormone use in women and their associations with dementia risk remain unclear.*

*What did the researchers do and find?*

- *Early and late menarche, younger age at first birth, and hysterectomy were associated with a greater dementia risk; ever been pregnant, ever had an abortion, longer reproductive span and later menopause were associated with a lower risk of all-cause dementia, after controlling for key confounders, using data from the UK Biobank.*
- *Hysterectomy, specifically hysterectomy without concomitant oophorectomy, or with a previous oophorectomy, were associated with greater dementia risk.*
- *Use of oral contraceptive pills was associated with a lower dementia risk.*
- *The U-shaped associations between number of children and dementia were similar for both sexes.*

*What do these findings mean?*

*Shorter cumulative exposure to endogenous estradiol is linked to greater dementia risk, although the risk variation in women was not due to biological factors associated with childbearing, because a similar U-shaped effect was found for number of children fathered in men. Putative mechanisms need to be evaluated in future studies.”*

**9. Main text: For in-text citations, please note reference numbers within square brackets, placed before the sentence punctuation, rather than superscript numbers. Where multiple references are listed, please do not include spaces within brackets.**

Thank you and apologies for the oversight. We have now updated the in-text citations so that they are consistent with the PLOS Medicine’s format.

**10. Introduction: Line 63: Please expand the description of the study’s objective to include that men were also included in the investigation.**

Thank you. We have updated the study’s objective to reflect this point. Please see page 5, line 96-98:

*“**In addition, we included men to compare the association between number of children fathered and the risk of all-cause dementia, with the association in female counterparts.”*

**11. Methods: Please ensure that the study is reported according to the STROBE guideline. Please add the following statement, or similar, to the Methods: "This study is reported as per the Strengthening the Reporting of Observational Studies in Epidemiology (STROBE) guideline (S1 Checklist)."**

Thank you for noting this. We have now included the following statement in the Methods section. Please see page 11, lines 222-223:

*“This study is reported as per the Strengthening the Reporting of Observational Studies in Epidemiology (STROBE) guideline (S1 Checklist)."*

**12. Methods: Did your study have a prospective protocol or analysis plan? Please state this (either way) early in the Methods section.**

**a) If a prospective analysis plan (from your funding proposal, IRB or other ethics committee submission, study protocol, or other planning document written before analyzing the data) was used in designing the study, please include the relevant prospectively written document with your revised manuscript as a Supporting Information file to be published alongside your study, and cite it in the Methods section. A legend for this file should be included at the end of your manuscript.**

**b) If no such document exists, please make sure that the Methods section transparently describes when analyses were planned, and when/why any data-driven changes to analyses took place.**

**c) In either case, changes in the analysis-- including those made in response to peer review comments-- should be identified as such in the Methods section of the paper, with rationale.**

The methods of the current study followed the study design and exposure selection used within of a previous paper from our group, which examined the associations between reproductive factors and cardiovascular diseases in the UK Biobank (Peters et al 2018).

Reference:

- Peters, S. A., & Woodward, M. (2018). Women’s reproductive factors and incident cardiovascular disease in the UK Biobank. *Heart*, *104*(13), 1069-1075.

Further, in response to peer review comments, additional analyses were conducted. We have made clear of these pre-specified plans and post-hoc updates on page 6, lines 100-112, in the methods section:

*“**Study design*

*The study design for the current study followed the main analysis plan from a previous study, which examined the associations between reproductive factors and cardiovascular diseases in the UK Biobank[14], with pre-specified subgroup analyses to assess effect modification.*

*The study design was further augmented by including broader selection and exposure categories, such as exogenous hormone use, and the timing of HRT in relation to menopause, as these are critical aspects to consider which may implicate the risk of dementia based on previous literature[4,8-13].*

*Post-hoc sensitivity analyses included death as a competing risk in estimating the associations between reproductive factors and dementia risk, given that dementia requiring a follow-up measurement which death may preclude. Combinations of reproductive factors, rather than examined in isolation, were also included in the ancillary analyses. Lastly, the analyses were reweighted according to the social structure of the population in general[15].”*

...And on page 10, lines 197-206:

*“We examined social deprivation in finer detail by grouping the Townsend Deprivation Score into fifths based on nationally derived cut-offs, given the heterogeneity observed in subgroup analysis by the pre-defined two-level SES, with the lowest fifth contained the 20% least socially disadvantaged, and the highest fifth contained the 20% most socially disadvantaged, with the interaction term fitted between the exposure of interest and Townsend fifths, and p for trend were presented. In addition, the results are weighted by the Townsend Deprivation Score: weighted HR are weighted according to equal weights of each stratified HR, to represent that the national Townsend fifths contain equal number of people. Whereas the unweighted HR represent the proportions across the fifths in the UK Biobank and calculated directly from overall Cox models.”*

**13. Methods: Line 101: Please provide more information on the Townsend Deprivation Index for determining SES and how this was categorized.**

Thank you for the suggestion. A recent publication from our group included information on social deprivation in the UK Biobank, please see:

- Woodward, M., Peters, S. A., & Harris, K. (2021). Social deprivation as a risk factor for COVID-19 mortality among women and men in the UK Biobank: nature of risk and context suggests that social interventions are essential to mitigate the effects of future pandemics. *J Epidemiol Community Health*.

We have now included more information on the Townsend Deprivation Index for determining social deprivation. Please see page 8, lines 152-157:

*“Social deprivation was determined from the Townsend Deprivation Index[18]. Townsend Deprivation Index measures area deprivation, comprised of four domains including about unemployment, car ownership, household overcrowding, and owner occupation, derived from the national census data, with higher scores indicate higher levels of social deprivation. The Townsend deprivation index was calculated for participant immediately prior to joining UK Biobank, based on the preceding national census output areas, in which the participant’s postcode is located.”*

Information on how we categorised Townsend Deprivation Index are included, please see page 10, lines 193-194:

*“Social deprivation (determined using the Townsend deprivation index at or below versus above the national median (-0.56)).”*

As well as for grouping Townsend Deprivation Score into fifths, please see page 10, lines 197-206:

*“**We examined social deprivation in finer detail by grouping the Townsend Deprivation Score into fifths based on nationally derived cut-offs, given the heterogeneity observed in subgroup analysis by the pre-defined two-level SES. The lowest fifth contained the 20% least socially disadvantaged, and the highest fifth contained the 20% most socially disadvantaged, with the interaction term fitted between the exposure of interest and Townsend fifths, and p for heterogeneity were presented. In addition, the results were weighted according to the social structure of the population in general: Weighted HR were calculated according to equal weights of each stratified HR by fifths of Townend Score, since the national Townsend fifths, by definition, contain an equal number of people.”*

**14. Results: For all the main results presented in the text, please note both 95% CIs and p values.**

95% confidence intervals, as well as p values, are now reported for main results presented in the text.

**15. Discussion: Please present and organize the Discussion as follows: a short, clear summary of the article's findings; what the study adds to existing research and where and why the results may differ from previous research; strengths and limitations of the study; implications and next steps for research, clinical practice, and/or public policy; one-paragraph conclusion.**

We have now updated our discussion, following the structure recommended.

**16. Discussion: Line 263: Please check if this should read “dendritic spine”.**

Thank you for noticing this typo, this has now been corrected. See page 26, line 377.

**17. Conclusions: Line 350: Here and throughout, please avoid language that implies causality, such as “This study highlights that the reproductive and endocrine milieu in women can have a significant impact on their dementia risk…” as this study is observational and therefore causality cannot be inferred.**

Thank you very much for pointing this out. We have carefully gone through the manuscript and toned down any language which may infer causality. Please see conclusion on page 30, lines 482-483, now it reads:

*“This study highlights that the reproductive and endocrine milieu in women may be involved in dementia risk.”*

**18. Tables and Supporting information tables: For all analyses presented, please include both adjusted and unadjusted analyses. Please report p values as well as 95% CIs.**

Both multiple-adjusted and unadjusted analyses are now included in tables and supporting information tables; p values as well as 95% CIs are reported.

**19. Table 1: Please note all abbreviations used in the legend (SES, HRT, etc).**

Sorry for the oversight, we have included all abbreviations used in the legend.

**20. Table 3: Please also present the unadjusted analyses. Please present p values in addition to 95% CIs.**

Thank you, we have now included the unadjusted analyses in Table 3. P-values and 95% Confidence intervals are presented.

**21. Table 4: Please also present the unadjusted analyses. Please define all abbreviations in the legend (HRT, BMI).**

Unadjusted analyses are now included, please see Supplementary Table 2 (S2 Table). All abbreviations included in the legend are also defined.

**22. S1 Table: The rates of dementia by number of children for men could be moved to the main text (the adjusted and unadjusted rates for women are presented in Table 2).**

Thank you, we have now moved this to the main text as suggested. Please see page 15, lines 249-253:

*“Among men, the multiple-adjusted rates of all-cause dementia per 10,000 person years (95% CIs) were: 8.65 (7.68, 9.62), 7.69 (6.63, 8.75), 7.95 (7.39, 8.51), 8.56 (7.70, 9.43), and 10.04 (8.67, 11.42), for those who fathered none, one, two, three, and four or more children, respectively. These rates were higher among men when compared with number of livebirths reported in women across all the categories (Table 2).”*

**23. Figure 1: Please describe each panel (A, B, C, etc) in the legend. Please also note in the legend the meaning of the line/shaded region.**

Thank you, we have now described each panel in the legend. The meaning of the line and shaded region are now described in the legend. Please see Figure legend on pages 36-37, lines 636-663.

*“Figure 1: Multiple-adjusted restricted cubic splines (with kernel density plots) showing HRs for dementia and reproductive factors.*

*HRT, Hormone Replacement Therapy.*

*The blue line represents the hazard function, blue shaded are represents the 95% confidence intervals. The shaded grey region represents the kernel density plot for the distribution of data.*

*After excluding the values from the top and bottom 2.5% of the distribution, with the median value being the reference. Splines adjusted for age, Townsend deprivation index, ethnicity, smoking status, systolic blood pressure, body mass index, diabetes, total cholesterol, antihypertensive drugs, lipids lowering drugs.*

*Panel A: Restricted cubic spline plot with multiple-adjusted hazard ratios (95% CI) for all-cause dementia associated with age at menarche.*

*Panel B: Restricted cubic spline plot with multiple-adjusted hazard ratios (95% CI) for all-cause dementia associated with age at first birth.*

*Panel C: Restricted cubic spline plot with multiple-adjusted hazard ratios (95% CI) for all-cause dementia associated with reproductive years.*

*Panel D: Restricted cubic spline plot with multiple-adjusted hazard ratios (95% CI) for all-cause dementia associated with age at natural menopause.*

*Panel E: Restricted cubic spline plot with multiple-adjusted hazard ratios (95% CI) for all-cause dementia associated with age at hysterectomy.*

*Panel F: Restricted cubic spline plot with multiple-adjusted hazard ratios (95% CI) for all-cause dementia associated with age at oophorectomy.*

*Panel G: Restricted cubic spline plot with multiple-adjusted hazard ratios (95% CI) for all-cause dementia associated with age started contraceptive pills.*

*Panel H: Restricted cubic spline plot with multiple-adjusted hazard ratios (95% CI) for all-cause dementia associated with age started HRT.*

*Panel I: Restricted cubic spline plot with multiple-adjusted hazard ratios (95% CI) for all-cause dementia associated with years using HRT.”*

**24. Figure 2: Please note in the legend that the** **bars represent the 95% CIs.**

Thank you, we have now updated the figure legend to include this information, please see page 37, lines 668-669:

*“The squares represent the hazard ratios, and the bars represent the 95% Confidence Intervals.”*

**25. References: Please use the "Vancouver" style for reference formatting (particularly journal title abbreviations), and see our website for other reference guidelines** [**https://journals.plos.org/plosmedicine/s/submission-guidelines#loc-references**](https://journals.plos.org/plosmedicine/s/submission-guidelines#loc-references)

We would like to apologise for this oversight. The “Vancouver” style for reference formatting is now adapted to the revised version of the manuscript.

**26. STROBE Checklist: Thank you for including the checklist. Please refer to locations in the text using section and paragraph numbers, rather than page numbers.**

Please see the updated STROBE Checklist submitted with the revision, we have referred to the locations in the text using section and paragraph numbers.

**Comments from the reviewers:**

**Reviewer #1: This paper investigates how reproductive factors are related to incident dementia The analyses are based on the UK biobank This a well written paper but there are several potential methodological issues that are not addressed**

**1. Firstly as authors are modeling age at dementia they need to make sure that the information used is accurate. In this case they use age at dementia based on the first time dementia is recorded and that includes death This is very likely to be biased as dementia is not fatal. This definition would need to be challenged in sensitivity analyses.**

Thank you for this comment. Based on this, we conducted additional sensitivity analyses, using multinomial regression which does not include the time-to-event, as per Cox Proportional Hazards Regression, and enables the competing risk of death to be considered. These models are compared as described below.

The methods describing these are included on pages 10-11, lines 210-219:

*“**We also constructed Fine and Gray competing risk models[20], and multinomial regression models[21], to compare with results from Cox Proportional Hazards Regression models, as sensitivity analysis. These methods will enable death to be accounted for as a competing risk for dementia, given that death may preclude dementia from occurring. The competing risk models which estimated sub-distribution Hazard Ratios, were conducted for all-cause dementia accounting for all-cause mortality as a competing risk, incorporating time to event data. For multinomial regression models, the odds ratios were produced, with outcomes specified as: 0) had neither dementia nor died by the last follow-up, 1) all-cause dementia by the last follow-up, 2) all-cause mortality preceding all-cause dementia by the last follow-up. Time to event is not specified in multinomial models.”*

These results are provided in Supplementary Table 4 (S4 Table). As shown, the results from the Cox models remained robust, with results from competing risk models and multinomial models broadly similar to the results from the Cox models. We included a paragraph highlighting this in the main text, please see page 23, lines 342-345:

*“**Results from the Competing Risk models (sub-distribution HRs) and Multinomial Regression models (Odds Ratios) which considered the competing risk of all-cause mortality, showed broadly similar results as the results from the Cox Proportional Hazards Regression models (HRs) (S4 Table).”*

**S4 Table: Multiple-adjusted Risk Ratios (95% confidence intervals) for the risk of dementia associated with reproductive factors, comparing Cox Proportional Hazards Regression (Hazard Ratios), Competing Risk Model (Hazard Ratios), and Multinomial Regression (Odds Ratios).**

| **Risk factors** | **Cox Regression Model ^a^**  **HR**  **(95% CI)** | **Fine and Gray Competing Risk Model**  **Sub distribution HR**  **(95% CI)** | **Multinomial Regression Model**  **OR**  **(95% CI)** |
| --- | --- | --- | --- |
| Age at menarche |  |  |  |
| <12 | 1.20 (1.03, 1.40) | 1.20 (1.03, 1.40) | 1.20 (1.03, 1.40) |
| 12 | 1.07 (0.92, 1.26) | 1.07 (0.91, 1.25) | 1.07 (0.92, 1.26) |
| 13 (ref) | - | - | - |
| 14 | 0.97 (0.83, 1.14) | 0.98 (0.83, 1.14) | 0.98 (0.83, 1.15) |
| >14 | 1.19 (1.02, 1.40) | 1.20 (1.03, 1.40) | 1.21 (1.04, 1.42) |
| Number of live births |  |  |  |
| 0 | 1.18 (1.02, 1.36) | 1.16 (1.01, 1.34) | 1.19 (1.03, 1.38) |
| 1 | 1.09 (0.93, 1.28) | 1.09 (0.93, 1.28) | 1.10 (0.94, 1.29) |
| 2 (ref) | - | - | - |
| 3 | 1.03 (0.91, 1.18) | 1.04 (0.91, 1.18) | 1.04 (0.91, 1.19) |
| 4 or more | 1.14 (0.96, 1.36) | 1.14 (0.96, 1.36) | 1.17 (0.99, 1.40) |
| Age at first live birth |  |  |  |
| <21 | 1.43 (1.18, 1.73) | 1.45 (1.20, 1.74) | 1.45 (1.20, 1.75) |
| 21-22 | 1.23 (1.02, 1.49) | 1.24 (1.02, 1.50) | 1.25 (1.03, 1.51) |
| 23-24 | 1.26 (1.05, 1.52) | 1.26 (1.05, 1.52) | 1.27 (1.05, 1.53) |
| 25-26 (ref) | - | - | - |
| 27-29 | 1.16 (0.96, 1.41) | 1.16 (0.96, 1.40) | 1.15 (0.95, 1.40) |
| >29 | 1.11 (0.91, 1.36) | 1.11 (0.91, 1.36) | 1.10 (0.90, 1.35) |
| Number of miscarriages |  |  |  |
| 0 (ref) | - |  |  |
| 1 | 0.90 (0.77, 1.04) | 0.90 (0.77, 1.04) | 0.89 (0.77, 1.04) |
| 2 or more | 1.02 (0.83, 1.26) | 1.02 (0.83, 1.26) | 1.03 (0.84, 1.28) |
| Number of stillbirths |  |  |  |
| 0 (ref) | - | - | - |
| 1 | 1.15 (0.88, 1.50) | 1.13 (0.86, 1.48) | 1.15 (0.88, 1.51) |
| 2 or more | 1.27 (0.66, 2.45) | 1.23 (0.64, 2.37) | 1.29 (0.66, 2.51) |
| Number of abortions |  |  |  |
| 0 (ref) | - | - | - |
| 1 | 0.97 (0.81, 1.16) | 0.98 (0.83, 1.18) | 0.95 (0.79, 1.14) |
| 2 or more | 0.34 (0.18, 0.64) | 0.36 (0.91, 0.67) | 0.33 (0.18, 0.62) |
| Reproductive years |  |  |  |
| <33 (ref) | - | - | - |
| 33-35 | 0.98 (0.79, 1.22) | 0.98 (0.79, 1.23) | 0.98 (0.78, 1.23) |
| 36-37 | 0.78 (0.62, 0.98) | 0.78 (0.62, 0.97) | 0.77 (0.61, 0.97) |
| 38-39 | 0.67 (0.54, 0.84) | 0.67 (0.53, 0.83) | 0.66 (0.53, 0.83) |
| 40-42 | 0.68 (0.55, 0.85) | 0.67 (0.54, 0.84) | 0.67 (0.54, 0.83) |
| >42 | 0.80 (0.63, 1.01) | 0.78 (0.62, 0.99) | 0.78 (0.61, 0.98) |
| Age at natural menopause |  |  |  |
| <47 | 1.32 (1.07, 1.62) | 1.33(1.08, 1.63) | 1.36 (1.10, 1.67) |
| 47-49 | 1.07 (0.91, 1.26) | 1.07 (0.85, 1.34) | 1.08 (0.86, 1.35) |
| 50 (ref) | - | - | - |
| 51-52 | 0.80 (0.68, 0.94) | 0.80 (0.64, 1.00) | 0.80 (0.64, 1.00) |
| 53-54 | 0.76 (0.62, 0.93) | 0.76 (0.59, 0.98) | 0.76 (0.59, 0.98) |
| >54 | 0.93 (0.80, 1.08) | 0.92 (0.75, 1.14) | 0.90 (0.73, 1.12) |
| Hysterectomy vs not | 1.12 (1.01, 1.25) | 1.12 (1.01, 1.25) | 1.12 (1.01, 1.25) |
| Oophorectomy vs not | 1.07 (0.92, 1.24) | 1.07 (0.92, 1.24) | 1.07 (0.92, 1.24) |
| Ever taken oral contraceptive pills | 0.80 (0.72, 0.88) | 0.81 (0.72, 0.90) | 0.77 (0.70, 0.86) |
| Age started oral contraceptive pills (per year) | 1.01 (1.00, 1.03) | 1.01 (1.00, 1.02) | 1.02 (1.00, 1.03) |
| Ever used HRT | 0.99 (0.90, 1.09) | 1.01 (0.91, 1.11) | 0.98 (0.89, 1.09) |
| Age started HRT (per year) | 0.96 (0.95, 0.98) | 0.96 (0.95, 0.98) | 0.96 (0.95, 0.98) |
| Duration of HRT use (per year) | 1.00 (0.98, 1.01) | 1.00 (0.98, 1.01) | 1.00 (0.98, 1.01) |

CI, Confidence Intervals; HR, Hazard Ratio; HRT, Hormone Replacement Therapy; OR, Odds Ratio.

Analyses were adjusted for age, Townsend index, ethnicity, smoking status, systolic blood pressure, body mass index, diabetes, total cholesterol, antihypertensive drugs, lipids lowering drugs.

^a^ These confidence intervals were calculated without the floating absolute risk, to provide comparable results to the competing risk models and the multinomial regression models.

**2. the authors report higher incidence of dementia in men than in women which is in disagreement with most reports. How do they explain that?**

The higher incidence of dementia in men than in women using the UK Biobank cohort has been reported in our previous publication:

- Gong, J., Harris, K., Peters, S. A., & Woodward, M. (2021). Sex differences in the association between major cardiovascular risk factors in midlife and dementia: a cohort study using data from the UK Biobank. *BMC medicine*, *19*(1), 1-11.

A recent paper included eight cohorts from the United States of America and Europe showed that the rates of dementia were similar for women and men (Wolter et al 2020). Another study from the CFAS study in the UK also showed a higher incidence in men than women (Matthews et al 2016). However, the evidence around sex differences in dementia incidence appear to vary across studies and highly dependent on geographical regions. Clear sex difference is more pronounced in the older age group, diverging around 75-79 years (Prince et al 2014). For reviewer’s information, please see the table included below, for age-specific sex ratios in the UK Biobank:

|  | **All participants** | | | **Incident dementia** | | |
| --- | --- | --- | --- | --- | --- | --- |
| **Age group at baseline** | Women | Men | % of women | Women | Men | % of women |
| **<50** | 64 647 | 53 186 | 54.9% | 46 | 43 | 51.7% |
| **50-54** | 43 242 | 33 051 | 56.7% | 73 | 76 | 49.0% |
| **55-59** | 50 733 | 40 041 | 55.9% | 183 | 202 | 47.5% |
| **60-64** | 66 062 | 55 335 | 54.4% | 519 | 595 | 46.6% |
| **65-69** | 47 369 | 46 109 | 50.7% | 1 002 | 1 234 | 44.8% |
| **≥70** | 1 187 | 1 235 | 49.0% | 43 | 52 | 45.3% |

As observed, the sex ratios across age groups at study baseline are similar in all participants, with higher percentage of women in younger age groups. Among those who had incident dementia, the proportions of women are slightly higher in younger age groups at study baseline.

References:

- Wolters, F. J., Chibnik, L. B., Waziry, R., Anderson, R., Berr, C., Beiser, A., ... & Hofman, A. (2020). Twenty-seven-year time trends in dementia incidence in Europe and the United States: The Alzheimer Cohorts Consortium. Neurology, 95(5), e519-e531.
- Matthews, F. E., Stephan, B. C., Robinson, L., Jagger, C., Barnes, L. E., Arthur, A., & Brayne, C. (2016). A two decade dementia incidence comparison from the Cognitive Function and Ageing Studies I and II. Nature communications, 7(1), 1-8.
- Prince, M et al (2014) Dementia UK: Update Second Edition report produced by King’s College London and the London School of Economics for the Alzheimer’s Society

**3. The authors use multiple indicators of reproductive life and this poses two problems: first a DAG would be useful to understand the underlying mechanisms that are investigated and this will help the reader to understand the analytical strategy, second the authors might consider correcting for multiple comparisons if they don't explain their strategy better.**

Thank you for these suggestions. These reproductive factors were chosen because of their association with cardiovascular diseases, highlighted in a previous paper from our group (Peters et al 2018). We did not have particular underlying mechanism in mind, but we aimed to determine whether these factors associated with cardiovascular diseases also associate with dementia. We prefer not to impose correction for multiple comparisons since this is only one paper, of ours, from amongst a large number using the same database, and there is no logical reason why such correction should be restricted to those in this paper alone. Instead, our policy is to mention the issue in Discussion, and we leave the reader to make their own interpretation. Further, we consider statistical significance as a secondary issue and interpreted our findings based on the effect size and clinical significance.

Reference:

- Peters, S. A., & Woodward, M. (2018). Women’s reproductive factors and incident cardiovascular disease in the UK Biobank. *Heart*, *104*(13), 1069-1075.

**4. the authors would need to take into account death as a competing event**

Thank you for the suggestion. Based on this comment, we have included additional sensitivity analyses taking the competing risk of mortality into account, using the Fine and Gray competing risk models, which considered time-to-event; and separately using the multinomial regression models, which ignored time-to-event. Please see the results in the supplementary material, where we compared the risk estimates using three different modelling strategies (Cox Proportional Hazards Regression, Fine and Gray Competing Risk Model, and Multinomial Regression), outlined further in response to your first comment. Noting that results were broadly similar across the modelling strategies.

**Reviewer #2: This is a very interesting and important manuscript examining reproductive risk factors in women and men using UK Biobank data. The authors report that several aspects of reproduction in women including age at menarche, age at menopause, hysterectomy, oral contraceptive use, and hormone replacement therapy are associated with risk of dementia. Further, there were U-shaped associations between number of children and risk of dementia for both women and men. Overall, the manuscript is well-written. My comments are enumerated below.**

**1.	A major concern is the lack of consideration of race/ethnicity. It is well known that age of menarche and menopause tend to occur earlier in women who are black as compared to white. Thus, race/ethnicity must be considered in the analyses. It should be reported in the tables and stratification is warranted.**

Thank you for this important consideration. The UK Biobank participants are predominantly white ethnicity (94.1%), the remaining ethnicities are predominantly Indian (1.2%), followed by Caribbean (0.9%), African (0.7%), Pakistani (0.4%), Chinese (0.3%) and other (2.4%). As such, there would be extremely limited power to consider stratification by ethnicity.

Ethnicity is now included in the covariate adjustment strategy, and we have updated all the models including ethnicity in the multiple adjustments. This is now described in the methods, please see page 9, lines 174-176:

*“Unadjusted, and multiple adjusted models included age at study entry, SES, ethnicity, smoking status, systolic blood pressure, BMI, history of diabetes mellitus, total cholesterol, antihypertensive drugs, and lipid-lowering drugs.”*

**2.	The Townsend deprivation index is included as a covariate, but education is not. Although these should be related, it is also important to assess the effect of the number of years of education separately. Women with age at first birth who were aged 24 or less were at greater risk of developing dementia - this could partially be influenced by education. Further, women with an abortion were at reduced risk of dementia - could this also be related to education?**

Thank you for this suggestion. We agree that it would be relevant to include education as a confounder in the relationship between reproductive factors and dementia. However, we don’t have education measures requested for our UK Biobank dataset and would not be able to obtain such information from UK Biobank before the submission deadline. We agree that the Census-based indices, like Townsend, and education are expected to be highly correlated (Woodward et al 1996), and deem it very unlikely that the results would be materially different if we were to adjust for years of education instead of for Townsend deprivation index.

Reference:

- Woodward M. Small area statistics as markers for personal social status in the Scottish heart health study. Journal of Epidemiology and Community Health 1996; 50: 570-76

**3.	Premenopausal bilateral oophorectomy, but not unilateral oophorectomy, has been associated with risk of dementia. Is it possible to determine whether the oophorectomy was unilateral or bilateral?**

Thank you very much for highlighting this interesting research question. Unfortunately, this was not collected by the UK Biobank.

**4.	There is no discussion of missingness. Presumably with this large of study, some data was missing. How was this handled? Were individuals excluded (in which case how did they compare to those included), or was missing data imputed?**

Thank you for this important comment. We conducted complete case analyses, as described on page 11, line 220:

*“All analyses were performed on complete case data...”*

For missing data, we have now reported the missingness for the variables of interest, included as a supplementary table. Please see supplementary table 1 (S1 Table) in the updated version of the supplementary material file, and mentioned on page 12, line 237-238:

*“**Missing data for each reproductive variable of interest were reported in the Supplementary (S1 Table).”*

**S1 Table: Number of missingness for each reproductive factor of interest in the UK Biobank.**

| **Risk factor** | **Women**  **(N=273 240)** | **Men**  **(N=228 957)** |
| --- | --- | --- |
|  | **N (%)** | **N (%)** |
| Age at menarche | 8 840 (3.2) | - |
| Ever been pregnant | 788 (0.3) | - |
| Number of children | 828 (0.3) | 4 271 (1.9) |
| Age at first live birth ^a^ | 390 (0.2) | - |
| Number of miscarriages ^b^ | 455 (0.5) | - |
| Number of stillbirths ^b^ | 264 (0.3) | - |
| Number of abortions ^b^ | 894 (1.0) | - |
| Reproductive years ^c^ | 32 153 (19.5) | - |
| Age at natural menopause ^c^ | 29 144 (17.6) | - |
| Hysterectomy | 1 000 (0.4) | - |
| Oophorectomy | 4 530 (1.7) | - |
| Ever taken oral contraceptive pills | 1 416 (0.5) | - |
| Age started oral contraceptive pills ^d^ | 8 179 (3.7) | - |
| Ever used HRT | 1 556 (5.7) | - |
| Age started HRT ^e^ | 10 677 (10.3) | - |
| Duration of HRT use ^e^ | 28 891 (27.8) | - |

HRT, Hormone Replacement Therapy.

^a^ Collected in women who indicated that they ever had given birth to more than one child (N = 184 876).

^b^ Collected in women who indicated that they ever had stillbirth, spontaneous miscarriage, or termination (N = 87 193).

^c^ Collected in women who indicated that their periods had stopped (N = 165 301).

^d^ Collected in women who indicated that they had taken the contraceptive pill (N = 220 344).

^e^ Collected in women who indicated that they had used HRT (N = 103 855).

**5.	What is meant by incident 'fatal' dementia? I have not heard this term before. Does this mean it was only reported on the death certificate but not the medical record?**

Thanks for the comment. Indeed, incident fatal dementia refers to dementia identified through the death records, but not recorded in the hospital episode records.

**6.	Was the duration of contraceptive use associated with risk of dementia?**

Thank you for this question. Unfortunately, information on duration of contraceptive pill use is not available, or derivable, from the UK Biobank.

**7.	Given HRT and contraceptive use was ascertained for women, could the authors also look at testosterone use for men?**

Thank for the suggestion. Information on HRT use was only reported by women in the UK Biobank.

**8.	Timing of HRT in relation to menopause is important. Is there information regarding when HRT was started in relation to menopause? Several studies of women who start HRT around the time of menopause do not report an increased risk of cognitive decline or dementia.**

Thank you for highlighting this. We have now included additional analysis where we examine the age at HRT initiation in relation to age at menopause, and modelled for the risk of dementia, included as a part of the Supplementary Materials Figure 2 (S2 Figure).

**S2 Figure: Multiple-adjusted hazard ratios (95% confidence intervals) for the risk of dementia associated with the timing of HRT use in relation to age at (natural or artificial) menopause in postmenopausal women.**

CI, Confidence Intervals; HRT, Hormone Replacement Therapy.

Analyses were adjusted for age, Townsend index, ethnicity, smoking status, systolic blood pressure, body mass index, diabetes, total cholesterol, antihypertensive drugs, lipids lowering drugs.

These results are described on page 24, lines 333-336:

*“Further, there was no evidence of dementia risk varied by the timing of HRT initiation in relation to menopause, although those who had menopause with unknown HRT initiation were at an increased risk of dementia compared to those who had menopause without HRT use (HR, 1.49 (1.31, 1.69), p<0.01) (S2 Figure).”*

**9.	Each of the reproductive factors were examined in isolation but that is not how they occur over the lifespan for women. It would be helpful to show the combination of factors or to put multiple in a model to determine which aspects might be most important. For example, is hysterectomy associated with dementia because it often co-occurs with bilateral oophorectomy and results in an earlier age at menopause?**

Thank you for this suggestion. Age at menopause was recorded for those who had natural menopause; age at surgical menopause was indicated by the age at hysterectomy or the age at oophorectomy. We have presented the results on the risk of dementia associated with hysterectomy and oophorectomy, in isolation (Table 2) but also in co-occurrence (S1 Figure). These results indicated that, those who only had hysterectomy had an increased risk of dementia, in the absence of oophorectomy (Hazard Ratio: 1.13 (1.00, 1.28)). For those who underwent both hysterectomy and oophorectomy, the timing of these procedures appear to be important, such that only those who had hysterectomy after oophorectomy were associated with an increased risk of dementia (Hazard Ratio: 2.35 (1.06, 5.23)). These were described on page 23, lines 315-318:

*“**Regarding the timing of hysterectomy and oophorectomy, in comparison to women who never underwent hysterectomy or oophorectomy, women who had hysterectomy after oophorectomy had an increased risk (HR, 2.35 (1.06, 5.23), p=0.04), while the other variations for the timing of the procedures did not appear to be associated with dementia risk (S1 Figure).”*

And on pages 26, lines 394-396:

*“**Moreover, consistent with findings on the timing of surgical menopause procedures in association with cardiovascular disease[14], our study also showed some evidence that there was a greater risk of dementia in those with hysterectomy with a previous oophorectomy.”*

From Table 1, the mean age at natural menopause was 50.3 years, whereas age at hysterectomy was 43.9 years and age at oophorectomy was 47.4 years. Indeed, on average, women who had hysterectomy and/or oophorectomy experience (surgically induced) menopause than those who had natural menopause.

In addition to examining hysterectomy and oophorectomy and their timing to each other in association with dementia, reproductive span which considered age at menarche and age at natural menopause in relation to menopause; timing of HRT initiation in relation to natural or artificial menopause. These measures considered several reproductive factors in combination, as well as their individual associations with dementia.

**S1 Figure: Multiple-adjusted hazard ratios for the risk of dementia associated with history and timing of hysterectomy and oophorectomy.**

CI, Confidence Intervals.

Analyses were adjusted for age, Townsend index, ethnicity, smoking status, systolic blood pressure, body mass index, diabetes, total cholesterol, antihypertensive drugs, lipids lowering drugs.

Further, our analyses sought to explore the individual associations between reproductive factors and dementia in the UK Biobank. This approach will enable us to use the maximum available data on each reproductive factor to estimate the associations. Your suggestion is an important consideration, as indeed multiple reproductive factors may be considered together. We have conducted a series of models to explore combinations of reproductive factors (S7 Table).

These were: (A) A consideration of pregnancy related factors: number of live births, stillbirths, miscarriages, and abortions, though there were no significant change in the model coefficients in this model compared with the individual associations. (B) A consideration of factors throughout the lifespan including age at menarche, parous vs not, hysterectomy and or oophorectomy, HRT use, contraceptive pill use.

Though there were no major differences in the conclusions of the reproductive factors in the combined model compared with when included individually, suggesting that there is no substantial effect of confounding.

**S7 Table: Multiple adjusted hazard ratios for comparing individual and combined associations between reproductive factors and dementia.**

| **Model 1** | **Individual associations**  **HR (95% CI)** | **Combined associations**  **HR (95% CI)** |
| --- | --- | --- |
| Per live birth | 0.98 (0.95, 1.02) | 1.02 (0.97, 1.07) |
| Per miscarriage | 1.01 (0.94, 1.08) | 1.01 (0.94, 1.08) |
| Per stillbirth | 1.10 (0.92, 1.31) | 1.09 (0.90, 1.31) |
| Per abortion | 0.82 (0.71, 0.94) | 0.82 (0.71, 0.94) |

**A.** A consideration of pregnancy related factors: number of live births, stillbirths, miscarriages, and abortions, though there were no significant change in the model coefficients in this model compared with the individual associations.

Analyses were adjusted for age, Townsend index, ethnicity, smoking status, systolic blood pressure, body mass index, diabetes, total cholesterol, antihypertensive drugs, lipids lowering drugs.

**B.** A consideration of factors throughout the lifespan including age at menarche, parous vs not, hysterectomy and or oophorectomy, HRT use, contraceptive pill use.

| **Model 2** | **Individual associations**  **HR (95%CI)** | **Combined associations**  **HR (95%CI)** |
| --- | --- | --- |
| Age at menarche |  |  |
| <12 | 1.20 (1.08, 1.34) | 1.19 (1.06, 1.32) |
| 12 | 1.07 (0.95, 1.20) | 1.08 (0.96, 1.22) |
| 13 (ref) | 1.00 (0.90, 1.11) | 1.00 (0.90, 1.12) |
| 14 | 0.97 (0.87, 1.09) | 0.98 (0.87, 1.10) |
| >14 | 1.19 (1.07, 1.34) | 1.18 (1.05, 1.32) |
|  |  |  |
| Parous vs not | 0.88 (0.77, 1.01) | 0.92 (0.80, 1.07) |
|  |  |  |
| No hysterectomy or oophorectomy (ref) | 1.00 (0.96, 1.06) | 1.00 (0.94, 1.07) |
| Hysterectomy without oophorectomy | 1.13 (1.00, 1.28) | 1.13 (1.00, 1.29) |
| Hysterectomy with oophorectomy | 1.09 (0.95, 1.26) | 1.11 (0.96, 1.29) |
|  |  |  |
| Ever used HRT | 0.99 (0.90, 1.09) | 1.00 (0.90, 1.11) |
| Ever taken oral contraceptive pills | 0.80 (0.72, 0.88) | 0.82 (0.73, 0.92) |

Analyses were adjusted for age, Townsend index, ethnicity, smoking status, systolic blood pressure, body mass index, diabetes, total cholesterol, antihypertensive drugs, lipids lowering drugs.

Methods were mentioned on page 9, lines 183-187:

*“**A series of models to explore combinations of reproductive factors were also constructed. These were: (A) A combination of pregnancy related factors: number of live births, stillbirths, miscarriages, and abortions; and (B) A combination of factors throughout the lifespan including age at menarche, parous vs not, hysterectomy and or oophorectomy, HRT use, contraceptive pill use.”*

These results were mentioned on page 24, lines 351-353:

*“**When reproductive factors in the combined model compared with models included these factors individually, there were no major differences, suggesting that there is no substantial effect of confounding (S7 Table).”*

**Reviewer #3: The overall significance of this paper is very high. Female-specific reproductive factors may explain sex differences in dementia prevalence. Further, the effects of oral contraceptives on dementia risk are unknown. Thus, the current study brings exciting new data to the field while appropriately addressing limitations of the study. Only minor revisions are suggested to consider additional factors in data interpretation and expand on a few discussion points.**

**Main Comments:**

**1. It is stated several times through the abstract, results, and discussion that for early menopause hysterectomy or oophorectomy that "women of lower SES had a greater dementia risk, but not for women of high SES." This statement could be misleading. Table 4 shows that even in high SES women that early menopause was still associated with increased risk (1.19 HR). Thus, it seems more accurate to convey that early menopause increased dementia risk, but that low SES women had increased risk relative to high SES women.**

Thank you and we agree with the suggestion, and we have updated the manuscript to highlight these changes, please see page 23, lines 319-320:

*“For early menopause, hysterectomy and oophorectomy, there was some evidence of heterogeneity by SES (p=0.05, p=0.01, p<0.01, respectively), such that women of relatively lower SES had increased dementia risk relative to women of higher SES (Table 4).”*

**2. Please discuss how HRT age of initiation could have played a role (if at all) in the SES difference? Did women with higher SES go into menopause earlier or later? Did they initiate HRT earlier or did a greater percentage of them undergo HRT? Could that have contributed to risk?**

Thank you for this excellent suggestion. We note that the characteristics by Townsend score fifths, on average, women of higher SES have menopause slightly later, and initiate HRT slightly later, compared with women of relatively lower SES, although these do not differ significantly. For the reviewer’s information, please see the table below, showing the baseline characteristics for age at menopause and HRT initiation by Townsend fifth.

| **Risk factors** |  | **Townsend fifths** | | | |
| --- | --- | --- | --- | --- | --- |
|  | **First**  **(<-2.938)** | **Second**  **(≥-2.938, <1.531)** | **Third**  **(≥-1.531, <0.170)** | **Fourth**  **(≥0.170, 2.448)** | **Fifth**  **(≥2.448)** |
| Age at menopause | 50.5 (4.3) | 50.4 (4.4) | 50.2 (4.5) | 50.0 (4.6) | 49.6 (4.8) |
| Age at HRT initiation | 47.8 (5.2) | 47.5 (5.3) | 47.3 (5.5) | 47.0 (5.7) | 46.4 (5.9) |

Some heterogeneity across Townsend score fifths were found for several reproductive factors: number of children in women, abortion, oophorectomy, and oral contraceptive pill use. These findings were broadly similar to the subgroup analysis by two-level SES at the national average cut-off.

In view of the results in Table 4 by two-level SES, we have reanalysed our main results by averaging across the social deprivation fifths (which are of equal size in the general population).

These results by Townsend score fifths are now included in our overall discussion and interpretation of the results, also presented in S5 Table. Please see page 23, lines 340-341:

*“There was some evidence of heterogeneity across Townsend score fifths (S5 Table), including number of children, abortion, oophorectomy, and oral contraceptive pill use in women.”*

**S5 Table: Multiple-adjusted Hazard Ratios (95% confidence intervals) for the risk of dementia associated with reproductive factors stratified by Townsend fifths.**

| **Risk factors** |  | **Townsend fifths** | | | | | |
| --- | --- | --- | --- | --- | --- | --- | --- |
|  | **First**  **(Least disadvantaged)**  **(<-2.938)** | | **Second**  **(≥-2.938, <1.531)** | **Third**  **(≥-1.531, <0.170)** | **Fourth**  **(≥0.170, 2.448)** | **Fifth**  **(Most disadvantaged)**  **(≥2.448)** | **P for heterogeneity across Townsend fifths** |
| Early menarche ^a^ vs. not | 1.23 (1.00, 1.52) | | 0.96 (0.72, 1.28) | 1.40 (1.05, 1.87) | 1.06 (0.76, 1.48) | 1.07 (0.81, 1.41) | 0.38 |
| Age at first live birth per year | 1.00 (0.98, 1.02) | | 0.99 (0.97, 1.02) | 0.98 (0.95, 1.01) | 0.99 (0.96, 1.03) | 0.96 (0.93, 0.98) | 0.18 |
| Each child ^b^ |  | |  |  |  |  |  |
| Women | 0.90 (0.83, 0.97) | | 0.96 (0.87, 1.06) | 0.94 (0.85, 1.05) | 1.06 (0.96, 1.17) | 1.06 (0.98, 1.13) | 0.01 |
| Men | 1.00 (0.93, 1.07) | | 1.00 (0.92, 1.10) | 1.05 (0.96, 1.14) | 1.03 (0.95, 1.12) | 1.04 (1.00, 1.09) | 0.83 |
| Stillbirth vs. not | 1.47 (0.96, 2.26) | | 1.03 (0.55, 1.95) | 0.74 (0.33, 1.66) | 1.16 (0.59, 2.27) | 1.16 (0.71, 1.87) | 0.65 |
| Miscarriage vs. not | 0.90 (0.72, 1.12) | | 0.92 (0.68, 1.23) | 0.82 (0.58, 1.15) | 0.93 (0.66, 1.33) | 1.11 (0.84, 1.46) | 0.70 |
| Abortion vs. not | 0.87 (0.62, 1.21) | | 0.66 (0.41, 1.06) | 1.03 (0.68, 1.55) | 0.99 (0.65, 1.51) | 0.82 (0.58, 1.16) | <0.01 |
| Early menopause ^c^ vs. not | 1.26 (0.95, 1.66) | | 1.28 (0.88, 1.86) | 1.36 (0.92, 2.02) | 1.90 (1.27, 2.85) | 1.78 (1.28, 2.48) | 0.32 |
| Hysterectomy vs. not | 0.94 (0.77, 1.14) | | 0.97 (0.75, 1.25) | 1.23 (0.94, 1.61) | 1.40 (1.05, 1.87) | 1.33 (1.05, 1.68) | 0.06 |
| Oophorectomy vs. not | 0.79 (0.58, 1.06) | | 0.75 (0.50, 1.11) | 1.31 (0.92, 1.87) | 1.83 (1.28, 2.62) | 1.23 (0.89, 1.69) | <0.01 |
| Oral contraceptive pill use vs. not | 1.01 (0.84, 1.22) | | 0.75 (0.59, 0.95) | 0.79 (0.61, 1.03) | 0.65 (0.49, 0.87) | 0.69 (0.55, 0.87) | 0.04 |
| HRT use vs. not | 0.93 (0.79, 1.11) | | 0.90 (0.72, 1.12) | 0.98 (0.77, 1.26) | 1.18 (0.89, 1.56) | 1.09 (0.87, 1.37) | 0.49 |

HRT, Hormone Replacement Therapy.

Analyses were adjusted for age, Townsend index, ethnicity, smoking status, systolic blood pressure, body mass index, diabetes, total cholesterol, antihypertensive drugs, lipids lowering drugs.

^a^ Early menarche was defined as age at first menstrual period before the age of 12 years.

^b^ Each livebirth in women and each child fathered in men.

^c^ Early menopause was defined as the permanent absence of menstrual periods before the age of 47 years.

**3.In Figure 1 it looks like some women started HRT very early - at 35 years of age and these were the women at highest risk (2.0 HR). Did these women also have premature menopause (either natural or surgical) that could have increased their risk? How was this accounted for in the interpretation of the data? For example, if women went through HRT early due to premature menopause (surgical or natural) then how was the risk due to early menopause vs. HRT separated statistically?**

This is a very interesting point, and we have since investigated this further. Please refer to Reviewer 2, Comment 8, where a similar issue was raised.

**Minor Comments:**

**1. In the abstract, the statement "although female biological factors involved in childbearing are unlikely to account for risk variation" was unclear on it is own. It would be helpful to say why.**

Thank you. Providing the similar associations for number of children and the risk of all-cause dementia between women and men, this finding indicates that the biological changes involved in pregnancy are unlikely to explain why this may account for the differences in dementia in risk in women, providing that we also observed a similar association in men, where the number of children fathered which gave a comparable U-shaped association. We have since made it clear in the abstract that the statement is based on the similar findings between women and men for the associations between number of children and dementia risk. Please see page 3, lines 52-53:

*“...there was a similar association between the number of children and dementia risk between women and men.”*

**2. In the final paragraph it is unclear what is meant by "Further, deprivation is likely to an important determinant of dementia". Deprivation of what? Estrogen?**

We would like to apologise for the oversight. We have now corrected this to “social deprivation”, as this is referring to the use of Townsend Deprivation Index. Please see page 29, lines 476-479:

*“Further, social deprivation is likely to be an important determinant of dementia risk and other aspects of women's health, given that the elevated dementia risk associated with early (natural and artificial) menopause and a greater number of children were more strongly associated among women of lower SES relative to women of higher SES.”*

**3. The final sentence it is unclear what the "Beijing Declaration" is. This ending to the manuscript felt odd.**

This mention of the declaration was intended to highlight the global health issues related to women’s health which our study may be relevant. We appreciate the feedback and have since removed this line in the conclusion.

**Reviewer #4:**

**This paper uses data from the UK Biobank to link reproductive factors and incident dementia.**

**I was asked for a statistical report and I interpret that to include all aspects of the design and conduct of the study.**

**Points of detail**

**Page 2 Something seems to have gone missing in the sentence starting `There was evidence'. In any event it needs the effect size and confidence interval.**

Thank you for highlighting this, we have now included numerical evidence along with the descriptions in the abstract. Please see pages 2-3, lines 45-48:

*“There was evidence of early menopause (HR, 1.75 (1.34, 2.29), p=0.05), hysterectomy (HR, 1.31 (1.09, 1.59), p=0.01), oophorectomy (HR, 1.39 (1.08, 1.78), p<0.01), and a greater number of children (HR, 1.06 (1.00, 1.13), p<0.01) associated with an increased risk of dementia among women of relatively lower SES, in relation to women of higher SES.”*

**Page 3 I feel that the authors should later tell us how they square the higher prevalence in women with the lower incidence rates which they found.**

Thank you for the comment. In the UK Biobank, women had a lower prevalence as well as lower incidence in comparison to men. As women tend to survive longer into older age, the prevalence of dementia tends to be higher in women than men, particularly in older age. Dementia onset might also be later in women. However, the UK Biobank is still relatively a young cohort, both the prevalence and incidence of dementia were lower in women than men in the UK Biobank. Please also see Reviewer 1, Comment 2, where a similar question was posed.

**Page 6 The finding of 263 prevalent cases is part of the results although the decision to exclude them is indeed part of the methods.**

Thank you for the comment. As the 263 prevalent cases at study baseline were excluded from all the analyses, these are included as part of the methods. Please see page 9, line 169:

*“The present analyses excluded participants with prevalent dementia at baseline (N=263).”*

**Figure 1 I am not sure which distribution was truncated at its extremes. Is this for the kernel plots? Incidentally in the version I received they are just a faint smudge and I almost missed them. Some of the age variables seem to show quite pronounced digit preference which perhaps limits their value.**

Thank you for this comment. We omitted the top and bottom 2.5% of the distribution (i.e. 5% of the total distribution excluded), as these extreme values are likely to be associated with unreliable estimates. These omissions were truncated for kernel plots as well as the splines, so that we can visualise more reliable relationships on the splines. This was made clear on pages 9-10, lines 188-191:

*“Multiple-adjusted restricted cubic splines (with kernel density plots) were constructed to assess the shape of continuous reproductive factors associated with dementia risk. The top and bottom 2.5% of the distributions, where precision is poor, were excluded, with the median value of the distribution taken as the reference.”*

In response to the last part of the comment, for certain variables, such as age at menarche, were reported as whole number for age in the UK Biobank.

See: <https://biobank.ctsu.ox.ac.uk/crystal/field.cgi?id=2714>

**Has the composition of hormonal contraception changed over time and if so does that matter for inferences?**

Thank you for the suggestion. As the hormonal contraceptive use was reported retrospectively, we do not have information on the change in hormonal contraception over time through a woman’s reproductive years prior to joining the UK Biobank. Unfortunately, the information related to hormonal contraceptive use was reported at study baseline (2006-2010) and it was repeated in 2012-2013 in 3.8% of the women only.

**Missing data**

**On page 7 we learn that the authors used complete data in their models. As far as I can see the amount of this is not reported, apologies if I missed it. However I think there are some clues in Table 1. If we look at miscarriages, for example, we find that the values given do not add up to 100% leaving approximately 16.8% unaccounted for. Other variables have similar rates of missing. Even if this is the same women each time it raises serious questions about the wisdom of using complete cases. Some attempt at imputation is called for here. We also need to see the number of missing for all variables as if 828 women cannot or will not tell us how many children they have had it does suggest many other variables have even more missing.**

Thank you. For women responded ‘prefer not to say’ to questions soliciting information on reproductive history, these were counted as missing in our study. We have now included a table outlining the missingness for the reproductive factor variables (please also refer to our response to reviewer 2, comment 4, where a similar issue around missing data was raised). Our analyses used complete case analyses, described on page 11, lines 220:

*“All analyses were performed on complete case data...”*

A supplementary table outlining the missingness for each reproductive factors of interest are reported in the supplementary Table 1 (S1 Table).

**Representativeness of UK Biobank**

**As the authors mention on page 23 the UK Biobank is known not to represent the UK population. In a different field (alcohol studies)** **Stamatakis et al. (2021) have shown that adjusting for the lack of representativeness made a profound difference. I think the authors would be well advised to consider similar supplementary analyses to reassure us. There must be national statistics on some of these variables. For instance the NHS website https://www.nhs.uk/conditions/stillbirth/ assures us that approximately 1 in 200 pregnancies (0.5%) ends in a still birth. Assuming they are independent this suggests that women with two pregnancies would have approximately a 1% chance of at least one stillbirth whereas about 2.6% of the women here reported at least one.**

Thank you for the important comment. The UK Biobank is indeed not representative of the UK general population, which is best characterised by the paucity of respondents in the lowest social deprivation group in the revised Table 1 (which now shows numbers by deprivation fifth rather than split at the median). But this does not necessarily mean that it is not suitable for examining associations between exposure measures with health outcomes (Woodward, 2014). Indeed, many authors (e.g., Fry et al, 2016; Batty et al, 2020) – including our own work (e.g. Millett et al, 2018; and Peters et al, 2020) - have shown that such results from the UK Biobank are similar, but with more precision due to the large sample size - to those found from bespoke cohort studies. The key issue is whether the social imbalance you point out has biased our findings. To address this issue, we have now reweighted our results (similar to Stamatakis (2021)) equally by the national deprivation fifths, as explained in our response to editor’s comment 1. The results appear in S6 Table.

These are included in the methods, see page 10, lines 202-206:

*“In addition, the results were weighted according to the social structure of the population in general: Weighted HR were calculated according to equal weights of each stratified HR by fifths of Townend Score, since the national Townsend fifths, by definition, contain an equal number of people.”*

And in results, page 24, lines 347-350:

*“... After weighting the estimates by Townsend fifths according to the national distribution, the pooled estimates were similar to those in the main analysis but with wider confidence intervals, caused by giving relatively higher weights to the fifths with the least precise estimates (S6 Table).”*

This was also included in the limitations section on page 30, lines 466-469.

*“After the results were weighted by Townsend fifths, there was extra imprecision in the weighted HR estimates for some exposures, which occurred where there is considerable difference in estimates (HR and standard errors) across the Townsend fifths; however, this may be a result of small numbers in some exposure categories.”*

Lastly, data from the NHS is almost always in tabular form, preventing statistical modelling of associations, and never has the richness of the UK Biobank data which allows for analyses of individual level associations adjusting for clinical, as well as socio-demographic, data. At best, they can only be used to compare prevalence with that in the UK Biobank, which is not what our study is concerned with – we study associations. They are also often out-of-date, as is the case with the data on stillbirths referred to.

**Form of relationship**

**Having shown us that the relationship between some variables is U-shaped the authors provide extensive coverage in the results and discussion of analysis per year which supposes a linear relationship. This is clearly inconsistent.**

**I know it is difficult to explain and interpret the interaction between a co-variate and a quadratic term but the linear results cannot stand.**

Thank you very much for this important consideration. We have since removed the emphasis in the text of the results and discussion, if the relationships showed on splines were not log linear.

**Summary**

**Some fairly major issues to sort out.**

**Michael Dewey**

**References**

**E Stamatakis, K B Owen, L Shepherd, B Drayton, M Hamer, and A E Bauman. Is cohort representativeness passe? Poststratified associations of lifestyle risk factors with mortality in the UK Biobank. Epidemiology, 32:179-188, 2021.**
