# Supplementary material for: Reproductive factors and the risk of incident dementia: A cohort study of UK Biobank participants
Source: PLoS Med. 2022 Apr 5;19(4):e1003955. doi: 10.1371/journal.pmed.1003955 (PMC8982865; doi:10.1371/journal.pmed.1003955)
Supplement: Attachment — Submitted filename: Response document_PMEDICINE-D-21-01592R2.docx [file pmed.1003955.s016.docx]

**Responses to Editor and referees:**

**Gong et al.** **Reproductive factors and the risk of incident dementia: a prospective cohort study of UK Biobank participants (PMEDICINE-D-21-01592R2)**

We thank the Editor and referees for considering our manuscript and providing us with the opportunity to revise and improve it. We have addressed all the editorial requests and responded to all comments provided by the editor and reviewer 4. We have addressed the comments point by point. Changes made in the manuscript are within quotation marks and are in italics. In addition, we provide a marked-up version of the manuscript, highlighting all changes and new material included in the manuscript.

**From the Academic Editor:**

**1. In terms of discussing the limitation of the representativeness of UK Biobank, please describe specific evidence on socioeconomic and other factors that might covary with each of the variables, e.g. abortion, stillbirth, early pregnancy, etc. It seems as if the adjustment for Townsend Deprivation Index and weighting according to social structure has in fact attenuated the statistical significance of associations. Please provide additional comment and interpretation for this result. Please discuss differential responses by deprivation and within different communities that could be related to some of the factors.**

Thank you for highlighting these issues. We have now addressed the comment in the limitations of the manuscript, please see page 30, lines 471-478:

*“**Socioeconomic position has been associated with several reproductive factors, such as age at first birth and parity.[47] After adjusting for Townsend Deprivation Index and weighting according to social structure in our study, the associations between reproductive factors and dementia attenuated.* *Given the limited representativeness of the UK Biobank, it would be prudent for future studies to understand the effect modification of socioeconomic positions on these reproductive factors and dementia risk in women, as some socioeconomic gradients can influence reproductive health, which may further exacerbate the risk of dementia, particularly in those that are more socially disadvantaged.”*

**Please discuss the limitations of the methods for determining dementia outcomes in terms of bias (including SES and age-related biases) and under-reporting.**

Thank you for the suggestion. Please see page 30, lines 478-482 for the mention of limitations of the methods for determining dementia outcomes.

*“**The adjudication of incident dementia in the UK Biobank used the date recorded in the death registry and hospital admission as the incidence date. Age and socioeconomic status, amongst other factors, might influence whether dementia is being recorded. It is also possible that some dementia cases were not picked up from hospital admission and death registry data, resulting in under-reporting of dementia cases.”*

**2. Response to reviewers: Please completely address the remaining concerns of Reviewer 4, including the points regarding multiple imputation as well as the inconsistencies with the interpretation of observed U-shaped relationships.**

Thank you. We have now completely addressed all the remaining concerns of Reviewer 4.

**3. Title: Please capitalize the first word of the subtitle, and please remove “prospective” from the title: “Reproductive factors and the risk of incident dementia: A cohort study of UK Biobank participants”**

Thank you for pointing this out, the subtitle has now been updated.

**4. Data Availability statement: Please omit the part of the statement indicating that UK Biobank resources are available from the authors, but do include the most direct link possible for data access requests (e.g.** [**https://www.ukbiobank.ac.uk/enable-your-research/apply-for-access**](https://www.ukbiobank.ac.uk/enable-your-research/apply-for-access)**) and also include any relevant (non-author) contacts (e.g.** [**access@ukbiobank.ac.uk**](mailto:access@ukbiobank.ac.uk)**).**

Thank you for the suggestion. Now the Data Availability statement has been updated to:

*“The UK Biobank resources are available upon reasonable request and can be accessed through applications on their website (*[*https://www.ukbiobank.ac.uk/enable-your-research/apply-for-access*](https://www.ukbiobank.ac.uk/enable-your-research/apply-for-access)*), and by contacting* [*access@ukbiobank.ac.uk*](mailto:access@ukbiobank.ac.uk)*.”*

**5. Abstract: Please combine the Methods and Findings sections into one section, “Methods and findings”.**

Thank you for the suggestion. This section has now been updated.

**6. Abstract: Line 37: Please change to “1866 dementia cases were recorded in women” if this is intended.**

Thank you for point this out, the word “cases” has now been added.

**7. Abstract: Please report exact p values, except where p<0.001.**

We have now reported all the exact p values.

**8. Author summary: What do these findings mean? Please format this section into 2-3 bulleted points.**

Please see the updated “Author Summary: What do these findings mean?” Please see page 4, lines 72-74:

- *Shorter cumulative exposure to endogenous estradiol is linked to greater dementia risk.*
- *Risk variation in women was not due to biological factors associated with childbearing, because a similar U-shaped effect was found for number of children fathered in men.”*

**9. Introduction: Line 95: Please avoid use of “effects” as this implies causality.**

Thank you for this important consideration. We have since updated the sentence, see page 5, lines 94-95, instead of using “effects”, the sentence now reads:

*“We assessed whether the associations between these factors and the risk of dementia vary by age, socioeconomic status, smoking status, and body mass index (BMI).”*

**10. Methods: Line 100: Please mention that there was no prospective analysis plan designed specifically for this study, though the framework for the design was drawn from the analyses carried out in a previous study.**

Thank you very much for this suggestion, please see page 6, lines 102-103, this section now reads:

*“No prospective analysis plan was specifically designed for the current study, though the framework for the design was drawn from the analyses carried out in a previous study, which examined the associations between reproductive factors and cardiovascular diseases in the UK Biobank[14], with pre-specified subgroup analyses to assess effect modification.”*

**11. Methods: Line 153: Please clarify if this should be “including information about unemployment…” or similar.**

Thank you very much for point this out, the word “information” is now included in the sentence, see page 8, lines 154-156, which now reads:

*“Townsend Deprivation Index measures area deprivation, comprised of four domains including information about unemployment, car ownership, household overcrowding, and owner occupation...”*

**12. Results: When reporting p values, please report the exact p value in the text, except please report p<0.001 where applicable. Please provide the exact p value where p<0.01.**

Thank you, the exact p values are now reported.

**13. Results: Line 330-331: Please revise to avoid casual implications “...no evidence of HRT duration affecting dementia risk…” and please avoid causal language throughout.**

We have now updated the sentence such that the causal language is now omitted, please see page 24, lines 331-332, with the sentence now reads:

*“...no evidence of HRT duration associated with dementia risk...”*

**14. Discussion: Line 381-383: Please clarify this sentence as the meaning is not clear. “The discrepancy in findings cannot be explained by the exclusion of women who reported hysterectomy or oophorectomy in the two studies reported opposite findings”**

Thank you for the comment. We have since clarified the sentence, please see page 26, lines 388-391:

*“**The discrepancy in findings between our study and the Gothenburg H70 and Rotterdam study[9,11] was not due to the exclusion of women who reported hysterectomy or oophorectomy in the Gothenburg H70 and Rotterdam study, as indicated in our sensitivity analyses.”*

**15. Discussion: Line 401: Please revise to avoid describing causal interpretations of the study findings.**

We would like to apologise for the oversight. We have checked the manuscript thoroughly and removed all causal language around any of the study findings.

**16. Discussion: Line 459: Please remove “...and the prospective design…” from the sentence.**

This part of the sentence has now been removed, see page 30, line 467.

**17. Page 32: Please remove the sections Availability of data and materials, Financial disclosure statement, Competing interests, and Author contributions from the main text of the manuscript. Please make sure the information is accurately and completely entered in the relevant fields of the manuscript submission system.**

The information is now removed from the main text of the manuscript and is entered in relevant fields of the manuscript submission system accurately and completely.

**18. Table 3, Table S2: Please report exact p values, unless p<0.001.**

Thank you, the exact p values for these analyses are now reported.

**19. STROBE Checklist: Thank you for including the checklist. Please revise, using only section and paragraph numbers to refer to locations within the text (e.g. Methods, paragraph 1). Please do not include page or line numbers.**

We have now revised the STROBE checklist, and only included section and paragraph numbers.

**20. S3 Table, S4 Table, S6 Table: Please report p values along with the 95% CIs for these analyses.**

Thank you, the p values along with the 95% CIs for these analyses are now reported for these supplementary tables.

**21. S5 Table, S2 Figure: Please report exact p values, unless p<0.001.**

Thank you, the exact p values for these analyses are now reported.

**22. S1 Figure and S2 Figure: Please also include a column with the HR, 95% CIs and p values from the unadjusted models.**

Thank you, a column with the HR, 95% CIs and p values from the unadjusted models are now included along with the multiple adjusted results, and these are now presented in table format. Please see S3 Table and S4 Table.

**23. Reference list: Please double check the reference formatting, and please see our website for additional guidance:** [**https://journals.plos.org/plosmedicine/s/submission-guidelines#loc-references**](https://journals.plos.org/plosmedicine/s/submission-guidelines#loc-references)

**For example, it seems as if the journal title for reference 7 should be Am J Obstet Gynecol.**

We would like to apologise for this oversight. The reference list has now been double checked thoroughly and updated according to the PLOS Medicine guideline.

**Comments from the reviewers:**

**Reviewer #2: The authors have addressed all of my comments.**

We would like to thank Reviewer 2 for your comments and consideration of our manuscript.

**Reviewer #3: All comments were addressed.**

We would like to thank Reviewer 3 for your comments and consideration of our manuscript.

**Reviewer #4:**

**This paper uses data from the UK Biobank to link reproductive factors and incident dementia.**

**I was asked for a statistical report and I interpret that to include all aspects of the design and conduct of the study. When I am asked to look at a re-submission which I had already reviewed on a previous submission I restrict myself on principle to commenting on how the authors have responded to my original comments. I do not review the article again from scratch unless it has been so changed as to amount effectively to a new submission.**

**I said:**

**The finding of 263 prevalent cases is part of the results although the decision to exclude them is indeed part of the methods.**

**Perhaps the authors misunderstood my point here. I agree that deciding to remove prevalent cases is part of the methods but the actual number involved was something that was only discovered during analysis and so is part of the results. I agree this is rather pedantic but it is still better to keep results all in the same place.**

We would like to thank Reviewer 4 for the detailed comments and apologise for the misunderstanding of the previous comment. We have now moved the information about excluding prevalent cases at the start of the results section. Please see page 9, line 170 for the updated text:

*“The present analyses excluded participants with prevalent dementia at baseline (N=263).”*

**I said:**

**On page 7 we learn that the authors used complete data in their models. As far as I can see the amount of this is not reported, apologies if I missed it. However I think there are some clues in Table 1. If we look at miscarriages, for example, we find that the values given do not add up to 100% leaving approximately 16.8% unaccounted for. Other variables have similar rates of missing.**

**Even if this is the same women each time it raises serious questions about the wisdom of using complete cases. Some attempt at imputation is called for here. We also need to see the number of missing for all variables as if 828 women cannot or will not tell us how many children they have had it does suggest many other variables have even more missing.**

**The authors have reported more detail about the numbers missing but have ignored my point about using some form of multiple imputation. I was expecting then either to provide the scientific reasons for rejecting this or to do it.**

Thank you for the suggestion. Many of the missing data are structurally missing, such that there is a logical reason for these to be missing, and hence imputation would not be inappropriate. For example, only those responded to the conditional question “Have you ever had any stillbirths, spontaneous miscarriages or terminations?”, would have information recorded for stillbirths, miscarriages and terminations (had abortions), which would only be applicable to those who have ever been pregnant. Please see the revised missingness table S1 Table, such that the percentage of missingness is calculated with those that are plausible to provide a response as the denominator.

**S1 Table: Number of missingness for each reproductive factor of interest in the UK Biobank.**

| **Reproductive factor** | **Women**  **(N=273 240)** | **Men**  **(N=228 957)** |
| --- | --- | --- |
|  | **N (%)** | **N (%)** |
| Age at menarche | 8 840 (3.2) | - |
| Ever been pregnant | 788 (0.3) | - |
| Number of children | 828 (0.3) | 4 271 (1.9) |
| Age at first live birth ^a^ | 867 (0.5) | - |
| Number of miscarriages ^b^ | 4 827 (2.1) | - |
| Number of stillbirths ^b^ | 4 636 (2.0) | - |
| Number of abortions ^b^ | 5 266 (2.3) | - |
| Reproductive years ^c^ | 32 628 (19.7) | - |
| Age at natural menopause ^c^ | 29 619 (17.9) | - |
| Hysterectomy | 1 000 (0.4) | - |
| Oophorectomy | 4 530 (1.7) | - |
| Ever taken oral contraceptive pills | 1 416 (0.5) | - |
| Age started oral contraceptive pills ^d^ | 8 657 (3.9) | - |
| Ever used HRT | 1 556 (5.7) | - |
| Age started HRT ^e^ | 12 511 (12.0) | - |
| Duration of HRT use ^f^ | 14 283 (16.3) | - |

HRT, Hormone Replacement Therapy.

^a^ Collected in women who indicated that they had ever given birth to more than one child (N = 184 876).

^b^ Collected in women who indicated that they had ever been pregnant (N = 231 352).

^c^ Collected in women who indicated that their periods had stopped (had natural menopause) (N = 165 301).

^d^ Collected in women who indicated that they had taken the contraceptive pill (N = 220 344).

^e^ Collected in women who indicated that they had ever used HRT (N = 104 133).

^f^ Collected in women who indicated that they had ever used HRT and not currently using HRT (N = 87 413).

We have previously shown that a complete case analysis is just as good as an imputed analysis when missingness is less than 10% (1), whilst others have shown that this is especially true when the sample size is large (2) and the hazard ratios are not (3), as in the current situation. As Table S1 shows, missingness only exceeded 10% for 4 of our 16 variables, reproduced below:

| Reproductive years | 32 628 (19.7) |
| --- | --- |
| Age at natural menopause | 29 619 (17.9) |
| Age started HRT | 12 511 (12.0) |
| Duration of HRT use | 14 283 (16.3) |

References:

- Barzi F, Woodward M. Imputations of missing values in practice: Results from imputations of serum cholesterol in 28 cohort studies. American Journal of Epidemiology 2004, 160(1):34-45.
- Little RJ. Missing data. In Encyclopedia of Biostatistics. Edited by: Armitage P, Colton T. New York: John Wiley and Sons; 1998:2622-2635.
- Demissie S, LaValley MP, Horton NJ, Glynn RJ, Cupples LA. Bias due to missing exposure data using complete-case analysis in the proportional hazards regression model. Statistics in Medicine 2003, 22(4):545-557.

We imputed missing data, using Multivariate Imputation by Chained Equations (MICE), with five iterations. The methods are included on page 11, lines 223-225:

*“Further, missing data were imputed using Multivariate Imputation by Chained Equations (MICE), with 5 iterations. The multiple-adjusted results from Cox regression models with imputed data were subsequently compared with the complete case analyses.”*

The results and interpretation are included as S10 Table and on page 25, lines 359-360:

*“After imputing missing data, the multiple-adjusted results were broadly similar to the results from the complete case analyses (S10 Table).”*

**S10 Table: Multiple-adjusted hazard ratios for the risk of dementia associated with reproductive factors in women, after imputed for missing data using Multivariate Imputation via Chained Equations (MICE), compared with complete case analyses.**

| **Reproductive factor** | **Imputed with MICE** | | **Complete case** | |
| --- | --- | --- | --- | --- |
|  | **Multiple-adjusted HR**  **(95% CI)** | **P-value** | **Multiple-adjusted HR**  **(95% CI)** | **P-value** |
| Age at menarche |  |  |  |  |
| <12 | 1.16 (1.05, 1.28) | 0.004 | 1.20 (1.08, 1.34) | 0.016 |
| 12 | 1.10 (0.99, 1.22) | 0.074 | 1.07 (0.95, 1.20) | 0.389 |
| 13 (ref) | 1.00 (0.91, 1.10) | - | 1.00 (0.90, 1.11) | - |
| 14 | 0.99 (0.89, 1.10) | 0.863 | 0.97 (0.87, 1.09) | 0.718 |
| >14 | 1.16 (1.04, 1.28) | 0.006 | 1.19 (1.07, 1.34) | 0.024 |
| Ever been pregnant | 0.86 (0.75, 0.99) | 0.036 | 0.85 (0.74, 0.98) | 0.026 |
| Number of live births |  |  |  |  |
| 0 | 1.11 (0.99, 1.26) | 0.079 | 1.18 (1.04, 1.33) | 0.027 |
| 1 | 1.01 (0.90, 1.14) | 0.855 | 1.09 (0.95, 1.25) | 0.276 |
| 2 (ref) | 1.00 (0.92, 1.09) | - | 1.00 (0.93, 1.08) | - |
| 3 | 1.04 (0.95, 1.15) | 0.406 | 1.03 (0.93, 1.15) | 0.641 |
| 4 or more | 1.12 (0.98, 1.29) | 0.106 | 1.14 (0.98, 1.33) | 0.132 |
| Parous vs not | 0.88 (0.78, 1.01) | 0.071 | 0.88 (0.77, 1.01) | 0.061 |
| Per live birth | 0.99 (0.96, 1.03) | 0.689 | 0.98 (0.95, 1.02) | 0.430 |
| Age at first live birth |  |  |  |  |
| <21 | 1.44 (1.28, 1.62) | <0.001 | 1.43 (1.26, 1.62) | <0.001 |
| 21-22 | 1.23 (1.08, 1.39) | 0.001 | 1.23 (1.08, 1.40) | 0.034 |
| 23-24 | 1.30 (1.15, 1.46) | <0.001 | 1.26 (1.12, 1.42) | 0.015 |
| 25-26 (ref) | 1.00 (0.87, 1.15) | - | 1.00 (0.87, 1.15) | - |
| 27-29 | 1.04 (0.91, 1.20) | 0.585 | 1.16 (1.02, 1.32) | 0.129 |
| >29 | 1.04 (0.87, 1.23) | 0.704 | 1.11 (0.96, 1.29) | 0.296 |
| Per additional year of age at first live birth | 0.97 (0.96, 0.98) | <0.001 | 0.98 (0.97, 1.00) | 0.006 |
| Number of miscarriages |  |  |  |  |
| 0 (ref) | 1.00 (0.95, 1.06) | - | 1.00 (0.94, 1.06) | - |
| 1 | 0.94 (0.83, 1.07) | 0.353 | 0.90 (0.78, 1.03) | 0.147 |
| 2 or more | 1.03 (0.85, 1.24) | 0.791 | 1.02 (0.83, 1.25) | 0.847 |
| Miscarriage vs not | 0.97 (0.86, 1.09) | 0.571 | 0.93 (0.82, 1.06) | 0.274 |
| Per miscarriage | 1.02 (0.96, 1.08) | 0.605 | 1.01 (0.94, 1.08) | 0.816 |
| Number of stillbirths |  |  |  |  |
| 0 (ref) | 1.00 (0.95, 1.06) | - | 1.00 (0.94, 1.06) | - |
| 1 | 1.17 (0.92, 1.49) | 0.196 | 1.15 (0.88, 1.49) | 0.312 |
| 2 or more | 1.39 (0.79, 2.45) | 0.257 | 1.27 (0.66, 2.45) | 0.472 |
| Stillbirth vs not | 1.20 (0.96, 1.51) | 0.116 | 1.16 (0.91, 1.49) | 0.234 |
| Per stillbirth | 1.11 (0.95, 1.31) | 0.185 | 1.10 (0.92, 1.31) | 0.319 |
| Number of abortions |  |  |  |  |
| 0 (ref) | 1.00 (0.94, 1.06) | - | 1.00 (0.94, 1.07) | - |
| 1 | 0.96 (0.82, 1.12) | 0.637 | 0.97 (0.82, 1.14) | 0.735 |
| 2 or more | 0.41 (0.24, 0.69) | <0.001 | 0.34 (0.18, 0.64) | <0.001 |
| Abortion vs not | 0.87 (0.74, 1.02) | 0.096 | 0.87 (0.73, 1.03) | 0.101 |
| Per abortion | 0.83 (0.73, 0.95) | 0.007 | 0.82 (0.71, 0.94) | 0.006 |
| Reproductive years |  |  |  |  |
| <33 (ref) | 1.00 (0.89, 1.13) | - | 1.00 (0.86, 1.17) | - |
| 33-35 | 0.93 (0.82, 1.05) | 0.262 | 0.98 (0.84, 1.15) | 0.878 |
| 36-37 | 0.79 (0.70, 0.89) | <0.001 | 0.78 (0.67, 0.92) | 0.031 |
| 38-39 | 0.67 (0.59, 0.76) | <0.001 | 0.67 (0.57, 0.79) | <0.001 |
| 40-42 | 0.69 (0.61, 0.78) | <0.001 | 0.68 (0.58, 0.79) | <0.001 |
| >42 | 0.68 (0.58, 0.78) | <0.001 | 0.80 (0.67, 0.95) | 0.056 |
| Age at natural menopause |  |  |  |  |
| <47 | 1.29 (1.15, 1.44) | <0.001 | 1.32 (1.15, 1.51) | 0.008 |
| 47-49 | 1.16 (1.02, 1.32) | 0.023 | 1.07 (0.91, 1.26) | 0.573 |
| 50 (ref) | 1.00 (0.88, 1.13) | - | 1.00 (0.86, 1.17) | - |
| 51-52 | 0.94 (0.83, 1.06) | 0.287 | 0.80 (0.68, 0.94) | 0.048 |
| 53-54 | 0.89 (0.77, 1.04) | 0.136 | 0.76 (0.62, 0.93) | 0.035 |
| >54 | 0.88 (0.78, 1.00) | 0.046 | 0.93 (0.80, 1.08) | 0.496 |
| Hysterectomy vs not | 1.11 (1.00, 1.22) | 0.053 | 1.12 (1.01, 1.25) | 0.039 |
| Oophorectomy vs not | 1.08 (0.94, 1.25) | 0.263 | 1.07 (0.92, 1.24) | 0.413 |
| Ever taken oral contraceptive pills | 0.79 (0.72, 0.88) | <0.001 | 0.80 (0.72, 0.88) | <0.001 |
| Age started oral contraceptive pills (per year) | 1.02 (1.00, 1.03) | 0.009 | 1.01 (1.00, 1.03) | 0.143 |
| Ever used HRT | 0.96 (0.87, 1.05) | 0.367 | 0.99 (0.90, 1.09) | 0.828 |
| Age started HRT (per year) | 0.96 (0.95, 0.98) | <0.001 | 0.96 (0.95, 0.98) | <0.001 |
| Duration of HRT use (per year) | 1.00 (0.98, 1.01) | 0.521 | 1.00 (0.98, 1.01) | 0.526 |

CI, Confidence Intervals; HR, Hazard Ratio; HRT, Hormone Replacement Therapy.

Analyses were adjusted for age, Townsend index, ethnicity, smoking status, systolic blood pressure, body mass index, diabetes, total cholesterol, antihypertensive drugs, lipids lowering drugs.

**I said:**

**Having shown us that the relationship between some variables is U-shaped the authors provide extensive coverage in the results and discussion of analysis per year which supposes a linear relationship. This is clearly inconsistent. I know it is difficult to explain and interpret the interaction between a covariate and a quadratic term but the linear results cannot stand.**

**There are still places where following the finding of a U-shaped relationship the authors then interpret it as linear. I did not do an exhaustive search but, for instance, on page 21 we read "Compared with those who had two children, the associations between the number of children and dementia were similar for women and men and were U-shaped (Figure 2). [. . . ] each additional child was associated with lower dementia risk in women [. . . ]. If the relationship is not linear then it is misleading to speak of the association of each additional child as non-linearity implies that is not constant across the range.**

We would like to apologise for the oversight. Thank you for pointing these out, and we have now checked the manuscript thoroughly and removed all the text where non-linear associations may have been interpreted as linear relationship.

**Still some issues to sort out.**

**Michael Dewey**
