# Supplementary material for: Reproductive factors and the risk of incident dementia: A cohort study of UK Biobank participants
Source: PLoS Med. 2022 Apr 5;19(4):e1003955. doi: 10.1371/journal.pmed.1003955 (PMC8982865; doi:10.1371/journal.pmed.1003955)
Supplement: Attachment — Submitted filename: Response document_PMEDICINE-D-21-01592R3.docx [file pmed.1003955.s017.docx]

**Responses to Editor and referees:**

**Gong et al.** **Reproductive factors and the risk of incident dementia: a prospective cohort study of UK Biobank participants (PMEDICINE-D-21-01592R3)**

We thank the Editor and referees for considering our manuscript and providing us with the opportunity to revise and improve it. We have addressed all the editorial requests in this revision. We have addressed the comments point by point. Changes made in the manuscript are within quotation marks and are in italics. We provide a marked-up version of the manuscript, highlighting all changes and new material included in the manuscript.

**Requests from Editors:**

**1. Throughout (Abstract Line 45-46, Line 52-53; Author Summary Lines 69-70 and 73-74; Results Lines 287-288; Discussion Lines 425-428, Conclusion Lines 497-499): A similar U-shaped association between number of children and the risk of dementia for both sexes is mentioned as a finding. The evidence in support of an overall relationship between number of children and risk of dementia was not entirely clear from the results. An apparent U-shaped pattern can be observed in Figure 2. The results seem to suggest a significant difference between women with no compared to 2 live births in the adjusted analyses, while there is no significant association with parity, or per live birth (Table 2). It is not completely clear, but from Figure 2, this may not be the case in men, and this would seem to suggest the relationship may differ between sexes to some extent. Also, Table 3 and Table S7 seem to suggest there may be different relationships between number of births and risk when SES/deprivation are examined, for women and men. Please clarify this throughout the manuscript, and revise conclusions as appropriate.**

Thank you for highlighting these issues. To clarify the message from the study, we have removed the log linear results (which were misleading) on per live birth for women in Table 3 and have now presented the results on number of children by category (0, 1, 2, 3, 4 or more) for women (number of live birth) and men (number of children fathered) directly side-by-side in figure 2 and in supplementary table 2 (S2 Table). We have now included the women-to-men ratio of hazard ratios, with p values, as suggested in comment 6, in supplementary table 2 (S2 Table). These results further support our conclusion of similar associations for the number of children and risk of dementia in both sexes, and as such, we believe that the original conclusion remains appropriate. We did, indeed, find that the effect of number of births differed by deprivation category, but the pattern of association remained unchanged. See also our response to your item 24.

**S2 Table: Unadjusted and multiple-adjusted hazard ratios, and women-to-men ratio of hazard ratios (95% confidence intervals) for the risk of dementia associated with number of children for women and men.**

|  | **Women** | | | | | **Men** | | | | | **Women-to-men** | | | |
| --- | --- | --- | --- | --- | --- | --- | --- | --- | --- | --- | --- | --- | --- | --- |
| **Number of children** | **No of events** | **Unadjusted HR**  **(95% CI)** | **P-value** | **Multiple-adjusted HR**  **(95% CI) ^a^** | **P-value** | **No of events** | **Unadjusted HR**  **(95% CI)** | **P-value** | **Multiple-adjusted HR**  **(95% CI) ^a^** | **P-value** | **Unadjusted RHR**  **(95% CI)** | **P-value** | **Adjusted RHR**  **(95% CI) ^a^** | **P-value** |
| 0 | 284 | 0.86  (0.75, 0.98) | 0.338 | 1.18  (1.04, 1.33) | 0.027 | 363 | 0.84  (0.74, 0.94) | 0.005 | 1.10  (0.98, 1.23) | 0.164 | 1.03  (0.88, 1.20) | 0.760 | 1.09  (0.92, 1.28) | 0.403 |
| 1 | 218 | 0.93  (0.79, 1.06) | 0.312 | 1.09  (0.95, 1.25) | 0.276 | 237 | 0.89  (0.77, 1.02) | 0.126 | 0.97  (0.84, 1.11) | 0.696 | 1.04  (0.86, 1.24) | 0.745 | 1.14  (0.94, 1.39) | 0.242 |
| 2 (ref) | 773 | 1.00  (0.93, 1.07) | - | 1.00  (0.93, 1.08) | - | 876 | 1.00  (0.93, 1.07) | - | 1.00  (0.93, 1.07) | - | 1.00  (0.91, 1.10) | - | 1.00  (0.90, 1.11) | - |
| 3 | 392 | 1.25  (1.15, 1.35) | <0.001 | 1.03  (0.93, 1.15) | 0.641 | 421 | 1.19  (1.10, 1.29) | 0.003 | 1.08  (0.97, 1.19) | 0.237 | 1.05  (0.91, 1.20) | 0.577 | 0.96  (0.83, 1.12) | 0.692 |
| 4 or more | 190 | 1.69  (1.55, 1.83) | <0.001 | 1.14  (0.98, 1.33) | 0.132 | 225 | 1.49  (1.36, 1.62) | <0.001 | 1.26  (1.10, 1.45) | 0.003 | 1.13  (0.93, 1.37) | 0.258 | 0.93  (0.76, 1.14) | 0.530 |

CI, Confidence Intervals; HR, Hazard Ratio; RHR, Ratio of Hazard Ratios.

^a^ Analyses were adjusted for age, Townsend index, ethnicity, smoking status, systolic blood pressure, body mass index, diabetes, total cholesterol, antihypertensive drugs, lipids lowering drug.

The statistical approach to calculate women-to-men ratio of hazard ratios is included on page 10, lines 191-192:

*“...and the interaction term between the number of children and sex was used to obtain the women-to-men ratio of hazard ratios (RHR) [20].”*

**2. Abstract: Line 27: Please change “equivocal” to “uncertain” in this sentence.**

Thank you, the suggested change has now been incorporated.

**3. Abstract: Line 29: Please revise to avoid the word “effects” as this may imply causality. We suggest: “...whether the associations vary by age, socioeconomic status…”**

Thank you, the suggested change has now been incorporated.

**4. Abstract: Line 40: We suggest referring to either >14 or >/= 15 consistently throughout the manuscript. In the results, >14 yrs is used.**

We would like to apologise for the oversight. ‘>14 years’ is now used consistently throughout the manuscript.

**5. Abstract: Please combine the “Methods and Findings” and “Results” sections into a single “Methods and Findings” section.**

Thank you. The section has now been updated.

**6. Abstract: Line 45-46: For this sentence, please report whether there is a significant association between number of children and dementia risk, provide the results (HR and 95% CIs, p values) to support the mentioned associations between number of children and dementia risk. “The U-shaped associations between the number of children and the risk of dementia were similar for both sexes,”**

Based on this comment, we have now included the women-to-men ratio of hazard ratios, along with the corresponding p-values, and included in Supplementary Table 2 (S2 Table).

Please see the updated text in the abstract describing these results on pages 2-3, lines 45-49:

*“**compared with those with two children, for those without children the multiple-adjusted HR ((95% CIs), p-value) was 1.18 (1.04, 1.33) (p=0.027) for women, and 1.10 (0.98, 1.23) (p=0.164) for men, the women-to-men ratio of HRs was 1.09 (0.92, 1.28) (p=0.403); for those with four or more children, the HR was 1.14 (0.98, 1.33) (p=0.132) for women, 1.26 (1.10, 1.45) (p=0.003) for men, women-to-men ratio of HRs was 0.93 (0.76, 1.14) (p=0.530).”*

**7. Abstract: Line 46-47: Please mention that this association did not reach statistical significance for early menopause.**

Thank you for pointing this out. This part of the sentence is now removed from the abstract given the p value for the interaction term between the two SES groups for early menopause did not reach statistical significance.

**8. Abstract: Line 48-50: We suggest also mentioning the representativeness of UK Biobank population as one of the main limitations of the study.**

Thank you. This has now been included in the main limitations in the abstract, see page 3, lines 52-54:

*“**Limitations the study include potential residual confounding, and self-reported measures of reproductive factors, as well as the limited representativeness of the UK Biobank population.”*

**9. Abstract: Line 52-53: “...****and there was a similar association between the number of children and dementia risk between women and men.” We suggest removing or clarifying this point to avoid misinterpretation (please see point #1 above).**

Thank you for the suggestion. Our updated results of women-to-men ratio of hazard ratios indicated no difference between the sexes for the number of children and dementia risk, the original conclusion remains and hence was not modified.

**10. Author summary: Line 59: We suggest “uncertain” rather than “equivocal” in this point.**

Thank you, the suggested change has now been incorporated.

**11. Author summary: Line 69: We suggest revising to: “The U-shaped associations between number of children and dementia appeared similar…”**

Thank you, the suggested change has now been incorporated.

**12. Author summary: Line 72: We suggest revising to “****In this study we observed that certain reproductive factors are associated with greater risk of dementia. Future work is needed to understand whether this is related to the fact that these factors may be associated with shorter cumulative exposure to endogenous estradiol.” or similar.**

Thank you, the suggested change has now been incorporated.

**13. Author summary: Line 73-74: Please see point #1 above. We suggest revising to: “****Findings suggest that risk variation in women may not be associated with factors associated with childbearing because a similar U-shaped pattern was observed between number of children fathered and dementia risk among men.” or similar.**

Thank you, the suggested change has now been incorporated.

**14. Throughout: In-text citations: Please separate the square brackets from the preceding word with a space.**

Thank you, the suggested change has now been incorporated.

**15. Introduction: Line 91: We suggest “uncertain” rather than “equivocal” in the sentence.**

Thank you, the suggested change has now been incorporated.

**16. Results: Line 248: Please define the values in parentheses at the first point of use in the text, e.g. rate (95% confidence interval).**

Thank you, the suggested change has now been incorporated.

**17. Results: Line 287-288: Please revise the sentence to: “****Compared with those who had two children, the associations between the number of children and dementia were similar for women and men and appeared to be U-shaped (Figure 2).” Please describe the HR with 95% CIs and p values for associations overall, and separately for men and women.**

Thank you, the suggested change has now been incorporated.

The sex-specific HR with 95% CIs and p-values, as well as the women-to-men ratio of hazard ratios with 95% CIs and p-values were presented in Supplementary Table 2 (S2 Table).

Please see the results described on page 22, lines 298-304:

*“**Compared with those who had two children, the associations between the number of children and dementia were similar for women and men and appeared to be U-shaped (Figure 2 and S2 Table): for instance, for those who had no children, the adjusted HR was 1.18 (1.04, 1.33) (p=0.027) for women, and 1.10 (0.98, 1.23) (p=0.164) for men, with the women-to-men ratio of HRs being 1.09 (0.92, 1.28) (p=0.403); for those who had four or more children, the adjusted HR was 1.14 (0.98, 1.33) (p=0.132) for women, and 1.26 (1.10, 1.45) (p=0.003) for men, with the women-to-men ratio of HRs being 0.93 (0.76, 1.14) (p=0.530).”*

**18. Discussion: Line 364: We suggest revising to: “...with dementia risk later in life.”**

Thank you, the suggested change has now been incorporated.

**19. Discussion: Line 384-385: We suggest: “...****estradiol has been shown to be correlated with neuronal dendritic spine density [22]...” or similar, as it seems the reference does not describe regeneration.**

Thank you, the suggested change has now been incorporated.

**20. Discussion: Line 408-409: We suggest revising to: “...****providing further support that early cessation of hormones may be associated with poorer cognitive outcomes.” or similar.**

Thank you, the suggested change has now been incorporated.

**21. Discussion: Line 424-425: Please clarify if this should be: “...while nulliparity and having two to four children showed similar associations compared to primiparous women…”**

Thank you for pointing this out, the suggested change has now been incorporated.

**22. Discussion: Line 425-428: It is not clear from the results whether a statistically significant association between number of children and dementia risk was identified for both men and women. We suggest tempering this sentence accordingly. “****In our study, the number of children was similarly associated with dementia risk for women and men. As such, the risk variation in women appears to be more related to social and behavioural factors involved in parenthood rather than biological factors associated with childbearing.”**

Thank you, the suggested change has now been incorporated.

**23. Discussion: Line 460: We suggest revising to: “...****do not support associations between HRT and dementia risk…” or similar.**

Thank you, the suggested change has now been incorporated.

**24. Discussion: Line 474: We suggest adding an additional sentence describing the limited representativeness of the UK Biobank, and a clear description of why this is important to consider in terms of interpretations of your results.**

Please see the updated text in the limitation section on page 31, lines 486-492:

*“**The limited representativeness of the UK Biobank population, which is a cohort predominantly of relatively healthy and affluent people of Caucasian ancestry, means that it is unlikely to produce reliable estimates of either the prevalence of female reproductive factors or the risk of dementia in the UK population at large. Our finding of an interaction between social deprivation and certain reproductive factors suggests that the main results for these factors need to be treated with caution, although any error in estimated effect size appears to be minor.”*

**25. References: Please update the reference information for Referenes # 40 and # 46.**

Thank you for pointing these out. The reference information (now references #41 and 47) have now been updated.

**26. Figure 2: Please provide the results from both adjusted and unadjusted analyses for men (e.g. similar to the results for women are provided in Table 3).**

Both adjusted and unadjusted analyses (HR, 95% CIs, and p-values) for the number of children fathered in men in association with risk of dementia are now provided, please see Supplementary Table 2 (S2 Table). These are presented with the HRs for women, and women to men ratio of HRs (95%CIs) for direct comparison.

**27. S9 Table: Please provide p values in addition to 95% CIs.**

The p-values are now added to the table in addition to 95% confidence intervals.

**Comments from Reviewers:**

**Reviewer #4: The authors have now addressed my remaining points.**

We would like to thank Reviewer 4 for comments and consideration of our manuscript.
